# Counterintuitive PM_2.5_ Increases During COVID-19 Lockdown in Ilo, Peru: Coastal Meteorology and Cardiovascular Implications

**DOI:** 10.3390/ijerph23020191

**Published:** 2026-01-31

**Authors:** José Antonio Valeriano-Zapana, Mario Román Flores-Roque, Leonel Alonso Paccosonco-Sucapuca, Yudith Milagros Cari-Cari, Daniel Álvarez-Tolentino, Alex Huaman De La Cruz

**Affiliations:** 1 Grupo de Investigación en Ciencias de la Atmósfera, Instituto de Investigación para el Desarrollo del Perú (IINDEP), Universidad Nacional de Moquegua (UNAM), Prolongación Ciudad Jardín s/n, Ilo 18611, Peru; 2 Laboratorio de Calidad de Aire, Escuela Profesional de Ingeniería Ambiental, Universidad Nacional de Moquegua (UNAM), Prolongación Ciudad Jardín s/n, Ilo 18611, Peru; lpaccosoncos@unam.edu.pe; 3 Área de Nutrición, Hospital Base II Moquegua, Seguro Social de Salud (EsSalud), Urbanización Capillune s/n, San Francisco, Moquegua 04301, Peru; 4 Escuela Profesional de Ingeniería Ambiental, Universidad Nacional Intercultural de la Selva Central Juan Santos Atahualpa, Jr. Los Cedros 141, La Merced 12615, Peru

**Keywords:** COVID-19, air quality, PM_2.5_, ozone, meteorological normalization, random forest, health impact assessment, cardiovascular mortality, coastal city, Peru

## Abstract

**Highlights:**

**Public health relevance—How does this work relate to a public health issue?**

- Air pollution by fine particulate matter (PM_2.5_) is a leading environmental risk factor for cardiovascular mortality worldwide, with the Global Burden of Disease 2021 attributing approximately 7.8 million deaths annually to ambient PM_2.5_ exposure.
- The COVID-19 pandemic created a natural experiment to assess air quality responses to emission reductions, yet evidence from Latin American coastal industrial cities remains critically underrepresented in the global literature.

**Public health significance—Why is this work of significance to public health?**

- Despite reduced anthropogenic activity during lockdown, PM_2.5_ increased by 34% during early reopening phases (16.9 vs. 12.6 µg/m^3^ baseline), while O_3_ more than doubled (+108%) in austral winter, demonstrating that coastal meteorology can counteract emission reduction benefits.
- Variance decomposition revealed that O_3_ variability was almost entirely meteorology-driven (98%), while PM_2.5_ and NO_2_ showed balanced contributions from meteorology and COVID-19 restrictions (~50% each), highlighting the critical need for meteorological normalization in air quality policy evaluation.

**Public health implications—What are the key implications or messages for practi-tioners, policy makers and/or researchers in public health?**

- The PM_2.5_ increase during lockdown corresponded to approximately 3 additional cardiovascular deaths per 100,000 population annually, emphasizing that counterintuitive air quality responses can translate into measurable health burdens requiring location-specific intervention strategies.
- Temporary emission reduction policies alone are insufficient to achieve health-protective air quality in coastal industrial cities; integrated strategies must account for local meteorological dynamics, marine boundary layer effects, and non-linear atmospheric chemistry.

**Abstract:**

The COVID-19 pandemic created a natural experiment to assess air quality responses to emission reductions, yet evidence from Latin American coastal industrial cities remains scarce. This study examined how meteorological variability modulated the effects of COVID-19 restrictions on air quality in Ilo, a medium-sized coastal industrial city in southern Peru (~67,000 inhabitants). We analyzed daily concentrations of PM_10_, PM_2.5_, NO_2_, O_3_, and SO_2_ across six pandemic phases (January–December 2020) using multiple linear regression, variance decomposition, and Random Forest models. A health impact assessment translated PM_2.5_ changes into cardiovascular mortality estimates using Global Burden of Disease 2021 coefficients. Despite reduced anthropogenic activity, PM_2.5_ increased by 34% during early reopening (May–June: 16.9 vs. 12.6 µg/m^3^ baseline), whereas NO_2_ decreased consistently (13–19%), SO_2_ declined up to 65%, and O_3_ more than doubled (+108%) in austral winter. Variance decomposition revealed that O_3_ variability was almost entirely meteorology-driven (98%), while PM_2.5_ and NO_2_ showed balanced contributions from meteorology and restrictions (~50% each). The PM_2.5_ increase corresponded to approximately 3 additional cardiovascular deaths per 100,000 population annually. Coastal meteorology can counteract emission reductions, generating counterintuitive air quality responses and underscoring the need for meteorological normalization in policy evaluation.

## 1. Introduction

Atmospheric pollution by fine particulate matter (PM_2.5_) is one of the leading environmental risk factors for global public health. Recent estimates from the Global Burden of Disease (GBD) 2021 indicate that ambient PM_2.5_ exposure is responsible for around 7.8 million deaths and over 230 million disability-adjusted life years (DALYs) worldwide, with the greatest burdens occurring in settings with lower socio-demographic development indices [1,2]. In the Latin America and Caribbean (LAC) region, cardiovascular diseases (CVDs) remain the primary cause of mortality and disability; in 2021, an estimated 22.2 million CVD DALYs were reported, of which about 12% were attributable to particulate matter pollution [3]. Despite this growing evidence base, comprehensive policy frameworks targeting urban transport-related air pollution in LAC are still less developed than in other regions [4].

The COVID-19 pandemic, declared a global health emergency by the World Health Organization on 11 March 2020, triggered unprecedented public health interventions affecting approximately 3.9 billion people across 157 countries through mobility restrictions and economic lockdowns [5,6]. These measures imposed substantial socioeconomic costs including deep economic contraction, unemployment, and disruptions in healthcare services, but simultaneously created a unique natural experiment to assess air-quality responses to rapid, large-scale emission reductions across multiple anthropogenic sectors [7,8]. By late March 2020, global daily CO_2_ emissions had declined by about 17% relative to 2019 baselines, and many cities reported marked decreases in PM_2.5_, PM_10_, nitrogen dioxide (NO_2_), sulfur dioxide (SO_2_), and carbon monoxide (CO) [9,10]. Systematic reviews and multi-city assessments consistently document substantial reductions in NO_2_ (typically 20–70%) and moderate decreases in PM_2.5_ and PM_10_ (7–60%), with particularly strong responses in some Asian and European cities [11,12,13,14,15,16,17]. In several locations, SO_2_ and CO levels also fell sharply [18,19]. Modeling studies suggest that short-term air-quality improvements during lockdowns likely prevented tens of thousands of premature deaths, particularly in regions with high baseline pollution [20,21,22,23].

However, emerging evidence shows that air-quality responses to COVID-19 measures were highly heterogeneous in space and time, challenging the assumption that emission reductions automatically translate into proportional concentration decreases [24,25,26,27,28,29,30,31]. Meteorology strongly modulates urban air pollution, and comparative studies using meteorologically normalized baselines have demonstrated that crude lockdown-related improvements were often overestimated by 30–50% [23,24,25,32]. Approaches such as multiple linear regression, generalized additive models, and machine learning methods (e.g., Random Forest) have been widely applied to separate meteorological from anthropogenic influences, highlighting that failing to account for weather can substantially bias estimates of intervention effects [23,24,25,26,33,34,35,36].

Ozone (O_3_) provides a clear illustration of atmospheric chemical complexity under changing emissions. Many cities reported 10–50% O_3_ increases during lockdowns despite large reductions in NO_x_ and other precursors [37,38]. This counterintuitive behavior reflects non-linear photochemistry in NO_x_-saturated, volatile organic compound (VOC)-limited regimes, where reduced NO emissions weaken the NO + O_3_ → NO_2_ + O_2_ titration pathway and allow ozone to accumulate [37,38,39]. Secondary PM_2.5_ formation can also respond non-linearly to changes in NO_x_, SO_2_, and VOC emissions, leading to episodes of severe haze even when primary emissions decline [40,41,42]. Overall, the COVID-19 period underscored the need for integrated, multi-pollutant strategies and for analytical frameworks that explicitly account for both atmospheric chemistry and meteorology [39,40,41,42].

In South America, lockdown-related air-quality changes have been less extensively documented than in Asia or Europe but show similarly complex patterns. In Lima, Peru, strict lockdown measures in March–April 2020 reduced PM_10_ and PM_2.5_ concentrations by roughly 40–58% and 31–43%, respectively, and lowered NO_2_ by nearly 50%, while O_3_ increased by 11–170%, consistent with a VOC-limited regime in a dense urban environment [43]. In Santiago, Chile, lockdowns produced significant reductions in PM_10_, PM_2.5_, and NO_x_, yet were accompanied by a 63% increase in O_3_ [44]. A satellite-based analysis of tropospheric NO_2_ in 17 Latin American cities reported substantial declines in columns over Lima (−47.5%), Santiago (−36.1%), São Paulo (−27%), Rio de Janeiro (−23%), Quito (−18.6%), and Bogotá (−17.5%) [45]. Beyond the pandemic, an Andean case study in municipalities near Bogotá showed that urbanization and industrial pressures can sustain elevated PM_10_ levels and hazardous trace-metal concentrations even during periods of reduced activity, with health-risk metrics for metals such as Cr (VI), As, and Co exceeding safety thresholds [46].

Despite the rapid expansion of COVID-19 air-quality literature, with over 2000 articles published in 2020–2021, largely concentrated in journals such as Science of the Total Environment, Aerosol and Air Quality Research, and Air Quality, Atmosphere & Health [14], several important knowledge gaps remain. First, geographic coverage is strongly biased toward Asian (≈65%) and European (≈18%) megacities, whereas medium-sized cities in developing regions, particularly in Latin America and Africa, remain underrepresented (≈5% and 3%, respectively) [14,47,48]. This imbalance raises questions about the generalizability of findings from well-studied megacities to smaller urban centers with distinct emission profiles, meteorological regimes, and socioeconomic contexts [29,30,31]. Second, coastal industrial cities constitute an understudied urban typology: marine boundary layer processes, sea-breeze circulations, port and shipping emissions, and hygroscopic sea-salt aerosols create air-quality dynamics that differ substantially from those in continental settings [32,49,50,51,52,53,54]. Conventional source-apportionment techniques can misattribute sea-salt contributions if they do not adequately distinguish marine sodium and chloride from anthropogenic sources [55,56]. Third, comparatively few COVID-19 air-quality studies translate observed concentration changes into explicit public-health metrics such as attributable mortality or morbidity, despite robust epidemiological literature showing that PM_2.5_ health effects extend below current WHO guideline values and that seemingly modest changes of 2–5 μg/m^3^ can yield measurable cardiovascular and respiratory impacts at the population level [57,58,59,60,61,62,63]. Finally, understanding how air pollution evolves during the post-lockdown recovery period is critical for policy design: analyses in China suggest that although PM_2.5_ levels rebounded when economic activity resumed, they remained below pre-pandemic baselines in cities that achieved a “green recovery”, combining economic growth with improved air quality [64].

This study addresses these gaps by analyzing air-quality responses to COVID-19 restrictions in Ilo, a medium-sized (≈67,000 inhabitants) coastal industrial city in southern Peru. Ilo hosts a large copper-smelting complex, port operations, and fishing activities, resulting in a mixed emission landscape combining industrial point sources, traffic, and marine aerosols [65]. Peru implemented one of the strictest national lockdowns globally, beginning on 16 March 2020 and followed by successive phases of mobility restrictions and partial reopening throughout the remainder of 2020 [6,66]. In Ilo, continuous monitoring at two stations (urban–industrial and residential) provides hourly measurements of PM_10_, PM_2.5_, NO_2_, O_3_, and SO_2_, together with meteorological variables, enabling an integrated assessment of anthropogenic and meteorological drivers.

The primary objective of this study is to quantify the relative contributions of COVID-19 restriction measures and meteorological variability to observed air-quality changes in Ilo during 2020. Specifically, we (1) characterize pollutant distributions and temporal patterns across six pandemic phases (Pre-Pandemic, Strict Lockdown, Phases 1–4); (2) examine meteorological controls and phase-stratified dispersion relationships; (3) partition explained variance between meteorological predictors (temperature, wind speed, relative humidity) and COVID-19 phases using variance decomposition via nested multiple regression and ANOVA F-tests; (4) evaluate non-linear relationships and variable importance using Random Forest models that relax linearity assumptions; and (5) translate PM_2.5_ changes into cardiovascular mortality estimates using concentration–response functions from Latin American and global cohorts [51,52,58,59,60,61,62,67,68,69,70].

We hypothesize that (H1) meteorological variability explains a substantial fraction of pollutant variance—especially for O_3_ and other secondary pollutants—making meteorological normalization essential for interpreting intervention effects; (H2) coastal meteorology and marine aerosols can lead to muted or counterintuitive particulate responses, including potential PM_2.5_ increases during lockdown; (H3) traffic-related NO_2_ exhibits robust decreases consistent with global observations, whereas SO_2_ shows marked reductions linked to smelter operations; and (H4) even moderate PM_2.5_ changes during lockdown periods translate into measurable cardiovascular mortality impacts at the population level.

This work makes four main contributions to the COVID-19 air-quality and environmental-health literature. First, it provides one of the first comprehensive analyses from a Latin American coastal industrial city, helping to address the geographic underrepresentation of medium-sized urban centers in global COVID-19 air-quality research [4,14,47,67]. Second, it applies a variance-decomposition framework combining nested multiple regression, ANOVA F-tests, and Random Forest models to disentangle meteorological and anthropogenic drivers of pollutant variability, building on recent advances in meteorological normalization techniques [24,34,35,36,71]. Third, it explicitly translates PM_2.5_ changes into cardiovascular mortality estimates, responding to the relative scarcity of studies that quantify the epidemiological consequences of COVID-19-related air-quality changes [57,58,59,60,61,62,63]. Fourth, by analyzing six pandemic phases over the full calendar year 2020, it moves beyond simple pre-/during-lockdown contrasts and captures the dynamic evolution of air quality as restrictions tightened and progressively relaxed [20,23,52].

## 2. Materials and Methods

### 2.1. Study Area

The study was conducted in the city of Ilo, located in Ilo Province, Moquegua Region, in southwestern Peru (17°38′40″ S, 71°20′43″ W). Ilo is a medium-sized coastal city with approximately 66,000 inhabitants and an arid coastal-desert climate, characterized by very low annual rainfall (~3.4 mm) and a mean annual temperature of about 19 °C [72]. The local economy is dominated by copper smelting and refining, port operations related to mineral exports, and industrial-scale fishing, together with associated urban activities, resulting in a complex mixture of industrial and urban emission sources [73]. This coastal industrial setting shares characteristics with other port cities where marine boundary-layer dynamics, sea-breeze circulations, and shipping emissions create distinct air quality conditions [32,53,54].

### 2.2. Study Period and COVID-19 Phase Definition

The COVID-19 pandemic began in December 2019 and the Peruvian government adopted the first restrictions in March 2020. The study period covers 1 January to 31 December 2020, encompassing the full spectrum of COVID-19 restriction phases. Data from 2019 were used exclusively for instrument stability verification and seasonal consistency checks. The pandemic period was divided into six phases based on official government decrees [66]:

- Pre-Pandemic (1 January to 15 March 2020), representing baseline conditions before mobility restrictions;
- Strict Lockdown (16 March to 3 May 2020), characterized by mandatory home confinement, suspension of non-essential activities, and curfew enforcement;
- Phase 1 (4 May to 4 June 2020), initial economic reactivation with limited activities;
- Phase 2 (5 June to 30 June 2020), progressive reopening of additional sectors;
- Phase 3 (1 July to 26 September 2020), broader economic reactivation; and
- Phase 4 (27 September to 31 December 2020), advanced reopening with most sectors operational.

### 2.3. Data Sampling and Measurement

**Figure 1:**
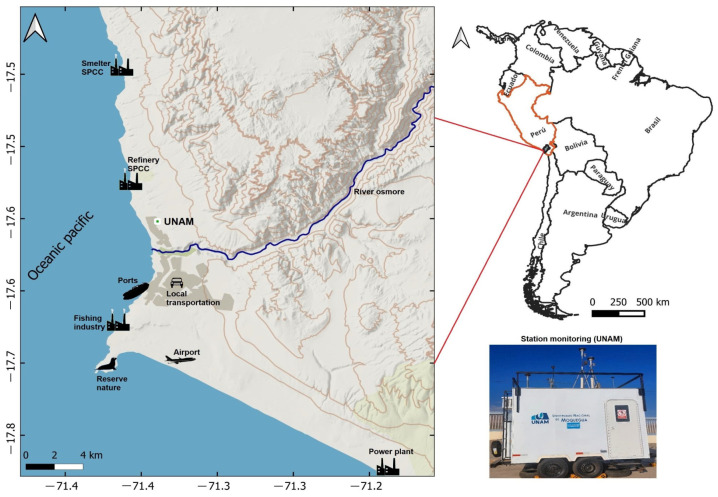
Geographic location of the study area and the fixed monitoring station at the National University of Moquegua (UNAM) in Ilo, Peru.

Hourly concentrations (µg/m^3^) of PM_10_, PM_2.5_, NO_2_, SO_2_ and O_3_ were measured from 1 January 2019 to 31 December 2020 at the fixed air-quality monitoring station of the National University of Moquegua (17°36′6.2″ S, 71°20′25.0″ W; Figure 1). Instrument types and reference methods for each pollutant are summarized in Table S1 (Supplementary Material). Concurrent meteorological variables—air temperature (°C), wind speed (m·s^−1^), wind direction (°) and relative humidity (%)—were recorded using a compact automatic weather station (Campbell Scientific^®^, Logan, UT, USA). The site is located in an open area, free from nearby direct emission sources and major obstructions such as tall buildings or trees.

### 2.4. Data Preprocessing and Quality Control

**Figure 2:**
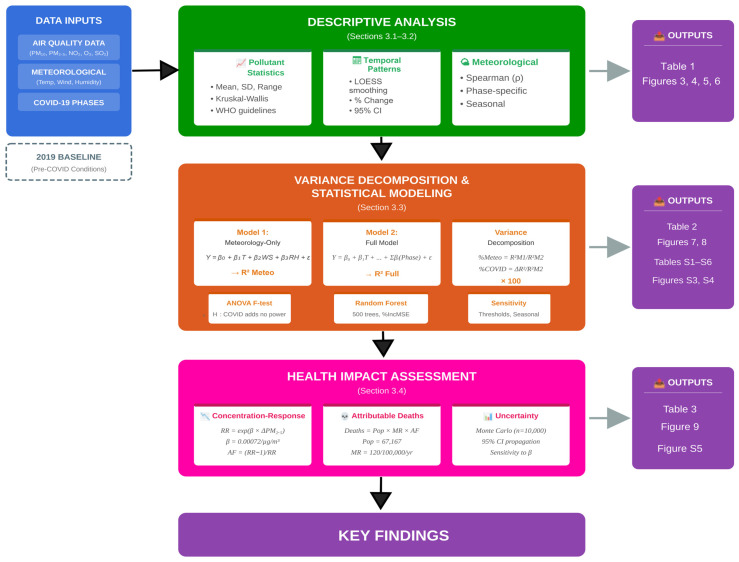
Methodological framework for the variance decomposition and health impact assessment of COVID-19 lockdown effects on air quality in Ilo, Peru (2019–2020). Blue boxes represent data inputs; green indicates descriptive analysis (Section 3.1 and Section 3.2); orange represents variance decomposition and statistical modeling (Section 3.3); pink denotes health impact assessment (Section 3.4). Purple boxes indicate outputs (tables and figures); dark blue box summarizes key findings. Dashed box shows the 2019 baseline reference period. Gray arrows indicate the analytical workflow, with thicker arrows showing the main analytical flow and thinner arrows pointing to outputs.

Hourly concentrations of PM_10_, PM_2.5_, NO_2_, O_3_, and SO_2_ and meteorological variables were retrieved from the UNAM monitoring station, focusing on the year 2020 (covering all COVID-19 restriction phases), while 2019 data were used only to verify instrument stability and seasonal consistency. Raw records had already undergone routine quality assurance by the network operator (calibration and range checks); we applied additional harmonized quality control, aggregating hourly values to daily means only when at least 18 valid hours (≥75% completeness) were available and excluding days below this threshold for the corresponding variable. This threshold is consistent with WHO recommendations for daily averaging [74] and has been applied in similar COVID-19 air quality studies [24,34,75]. The COVID-19 calendar was encoded as a six-level factor according to the phases defined in Section 2.2, and continuous meteorological predictors were mean-centered and standardized prior to modeling to improve numerical stability and comparability of regression coefficients. The overall analytical framework integrating data preprocessing, statistical modeling, and health impact assessment is summarized in Figure 2. This methodological workflow illustrates the sequential stages from raw data inputs through variance decomposition to final health impact quantification, providing a roadmap for the analyses described in the following sections.

### 2.5. Statistical Analysis Framework

All statistical analyses were performed in R version 4.5.1 [76] using RStudio as the integrated development environment. The analytical framework combined descriptive statistics, multiple linear regression with variance decomposition, Random Forest models, and sensitivity analyses to disentangle the relative contributions of COVID-19 restriction periods and meteorological variability to air pollutant concentrations. This approach follows methodological advances in meteorological normalization demonstrated in recent COVID-19 air quality studies [24,34,35,36,71].

#### 2.5.1. Descriptive Statistics

Descriptive statistics (mean, standard deviation, median, range) were calculated for each pollutant by pandemic phase. Distribution normality was assessed using Shapiro–Wilk tests and Q–Q plots. Differences in pollutant concentrations across phases were evaluated with Kruskal–Wallis tests, followed by Dunn’s post hoc tests with Benjamini–Hochberg correction when appropriate (α = 0.05). Temporal trends were visualized using locally weighted scatterplot smoothing (LOESS, span = 0.3) to capture non-linear patterns while limiting overfitting [77]. Spearman rank correlation coefficients (ρ) were computed to characterize associations between pollutants and meteorological variables, accounting for non-linear relationships and outliers.

Observed concentrations were compared against the 2021 World Health Organization Air Quality Guidelines [74]: PM_10_ = 45 µg/m^3^ (24 h), PM_2.5_ = 15 µg/m^3^ (24 h), NO_2_ = 25 µg/m^3^ (24 h), O_3_ = 100 µg/m^3^ (8 h peak), and SO_2_ = 40 µg/m^3^ (24 h). Exceedance frequencies were calculated for each pollutant and phase.

#### 2.5.2. Multiple Linear Regression Models

To quantify the independent effects of COVID-19 restrictions on air quality while controlling for meteorological confounders, we fitted nested multiple linear regression models for each pollutant, allowing explicit partitioning of variance between competing explanatory factors [78]. This nested model approach enables formal statistical testing of whether COVID-19 restriction periods explain additional variance beyond meteorological variability, as recommended by recent comparative methodological studies [35,36].

Model 1 (Meteorology-Only)

*Y* = *β*_0_ + *β*_1_(*Temperature*) + *β*_2_(*WindSpeed*) + *β*_3_(*Humidity*) + *ε* (1)

Model 2 (Full Model with COVID-19 Periods)

*Y* = *β*_0_ + *β*_1_(*Temperature*) + *β*_2_(*WindSpeed*) + *β*_3_(*Humidity*) + *Σβ_i_*(*Period_i_*) + *ε* (2)

where Y denotes daily pollutant concentration (µg/m^3^), Temperature (°C), Wind Speed (m·s^−1^) and Humidity (%) are continuous meteorological predictors, and Period_2–6_ are binary indicators for pandemic phases (reference: Pre-Pandemic). The error term ε is assumed to be normally distributed with constant variance. Meteorological predictors were selected based on their established roles in dispersion and atmospheric chemistry: temperature modulates photochemical reaction rates and boundary layer stability, wind speed controls dilution and transport, and humidity influences gas-to-particle conversion and wet deposition [39]. These meteorological variables have been identified as key predictors in Random Forest-based meteorological normalization studies across diverse urban settings [34,36,54,71].

Model assumptions were checked using standard diagnostics: residuals versus fitted values (linearity and homoscedasticity), normal Q–Q plots (residual normality), scale–location plots (variance homogeneity) and residuals versus leverage (influential observations). Variance inflation factors (VIFs < 5) confirmed absence of problematic multicollinearity among predictors [79].

#### 2.5.3. Variance Decomposition and Effect Quantification

The relative contributions of COVID-19 restrictions versus meteorological variability were quantified through incremental *R*^2^ comparison between nested models. The coefficient of determination (*R*^2^) represents the proportion of variance in pollutant concentrations explained by predictor variables [80]. This variance decomposition approach has been validated against more complex machine learning methods and shown to provide robust estimates of meteorological versus anthropogenic contributions [35].

Meteorological contribution

*R*^2^ *Meteo* = *R*^2^ *Model*_1_ (3)

COVID-19 contribution

*R*^2^ *COVID* = *R*^2^ *Model*_2_ − *R*^2^ *Model*_1_ (4)

Relative percentage contributions

%*Meteo* = (*R*^2^ *Meteo*/*R*^2^ *Model*_2_) × 100 (5)

%*COVID* = (*R*^2^ *COVID*/*R*^2^ *Model*_2_) × 100 (6)

The statistical significance of COVID-19 effects beyond meteorological variability was assessed using ANOVA F-tests comparing nested models [81]. The null hypothesis that COVID-19 periods provide no additional explanatory power beyond meteorology was tested at α = 0.05. Heteroscedasticity-robust standard errors [82] were computed to ensure valid inference when variance homogeneity assumptions were violated. Percentage changes in pollutant concentrations relative to the Pre-Pandemic baseline were calculated with 95% confidence intervals derived from standard errors of the mean, providing uncertainty estimates for observed reductions [83]:

Percentage change

%*Change_i_* = [(*Mean_i_* − *Mean PrePandemic*)/*Mean PrePandemic*] × 100 ± 1.96 × *SE diff* (7)

#### 2.5.4. Random Forest Analysis

To complement parametric regression and assess non-linear relationships, Random Forest (RF) models were implemented for each pollutant using the randomForest package [84,85]. RF is an ensemble machine learning technique that constructs multiple decision trees through bootstrap aggregation and provides robust variable importance measures [86]. RF-based meteorological normalization has been shown to outperform linear methods in capturing synoptic-scale variations, particularly when meteorological changes dominate pollutant variability [35]. Recent studies have demonstrated the utility of RF for COVID-19 air quality assessments in cities ranging from megacities to medium-sized urban areas [34,36,75].

RF models included the same predictors as linear models (temperature, wind speed, humidity, and pandemic period as categorical factors). Each model was trained with 500 trees (ntree = 500), with mtry set to the default value of *p*/3 for regression (where p = number of predictors) and a minimum node size of 5 observations. A random seed (set.seed = 123) was used to ensure reproducibility [87].

Out-of-bag (OOB) error rates quantified prediction accuracy without requiring separate validation sets, as each tree is trained on a bootstrap sample (~63% of data) and validated on remaining observations [85]. Variable importance was assessed using two metrics: (1) percent increase in mean squared error (%IncMSE) when a variable is randomly permuted, indicating prediction degradation when that variable is unavailable, and (2) total decrease in node impurities (IncNodePurity) from splits on each variable, measuring contribution to model accuracy [88]. Higher values indicate greater importance.

#### 2.5.5. Sensitivity Analyses

Several sensitivity analyses were performed to assess the robustness of the results: (1) alternative temporal aggregation, by repeating the analyses with different minimum hourly data thresholds for daily averages (≥12, ≥18 and ≥20 valid hours); (2) seasonal stratification, by re-fitting the models separately for austral summer (December–February) and winter (June–August); (3) outlier treatment, by excluding observations exceeding 3 standard deviations from the mean; and (4) alternative meteorological controls, by extending the models to include atmospheric pressure and solar radiation to check that meteorological confounding was adequately controlled. These sensitivity approaches follow recommendations from recent methodological comparisons of COVID-19 air quality studies [24,35,75].

### 2.6. Health Impact Assessment

To quantify the public health implications of PM_2.5_ variations observed during different epidemiological periods, we applied the concentration–response function (CRF) model recommended by the World Health Organization [74]. This methodological approach allows estimation of changes in attributable mortality associated with modifications in atmospheric pollutant concentrations [58,62]. Similar health impact assessments have been conducted for COVID-19 air quality changes in multiple countries, demonstrating the applicability of this approach to pandemic-related exposure variations [63,64].

#### 2.6.1. Concentration–Response Model

We implemented the standard log-linear model without threshold [74], expressed as:

Relative Risk

*RR* = *exp*(*β* × Δ *PM*_2.5_) (8)

where RR represents relative risk, β is the risk coefficient per unit increase (1 μg/m^3^) in PM_2.5_, and ΔPM_2.5_ corresponds to the change in mean concentration relative to the pre-pandemic reference period [89]. The attributable fraction (AF) was calculated as:

Attributable Fraction

*AF* = (*RR* − 1)/*RR* (9)

Annual attributable deaths were estimated as:

Attributable Deaths

*Deaths* = *Population* × *Baseline_Mortality_Rate* × *AF* (10)

following established methods in environmental health impact assessment [90].

#### 2.6.2. Epidemiological Parameters

We adopted a concentration–response coefficient of β = 0.00072 per 1 µg/m^3^ increase in PM_2.5_ (95% CI: 0.00059–0.00085) for cardiovascular mortality in adults, as reported by the Global Burden of Disease 2021 systematic analysis [2]. The GBD 2021 coefficient was selected as the primary estimate for three reasons: (1) it represents the most current meta-analytic synthesis, incorporating data through 2021 and methodological refinements in exposure assessment; (2) it has been validated for application across diverse regions, including Latin America, where recent analyses have employed this coefficient to estimate PM_2.5_-attributable cardiovascular burden [3,4]; and (3) it provides a conservative estimate compared to alternative sources (e.g., Pope et al. [91]: β = 0.00080; WHO 2021 [74]: β = 0.00076), reducing the risk of overestimating health impacts. The GBD 2021 coefficient represents a ~5% reduction from the GBD 2019 estimate (β = 0.00076), reflecting refinements in exposure assessment and outcome ascertainment [2]. To evaluate the sensitivity of our results to this choice, we repeated the health impact calculations using alternative β values from Burnett et al. [58], Pope et al. [91], and the WHO Air Quality Guidelines [74] (see Supplementary Figure S5). The baseline cardiovascular mortality rate was set at 120 deaths per 100,000 inhabitants per year, based on official statistics for the Moquegua region from the Peruvian Ministry of Health [92].

#### 2.6.3. Population Estimation

The exposed population was estimated at 67,167 inhabitants based on the 2017 National Census [72], updated using demographic projections for 2019–2020. A homogeneous exposure of this population to ambient PM_2.5_ concentrations measured at the monitoring station was assumed, acknowledging this as a standard limitation of ecological exposure–response studies [93]. Although cardiovascular mortality risk is predominantly concentrated in adults aged ≥30 years (approximately 68% of the population), we applied the concentration–response function to the total population, consistent with the GBD 2021 comparative risk assessment methodology that estimates population-level attributable burden [2].

#### 2.6.4. Comparative Periods

The health impact analysis was conducted across six COVID-19 periods: Pre-Pandemic (1 January–15 March 2020), Strict Lockdown (16 March–3 May 2020), and four progressive reopening phases (Phase 1: 4 May–4 June; Phase 2: 5–30 June; Phase 3: 1 July–26 September; Phase 4: 27 September–31 December 2020). For each period, the change in mean PM_2.5_ (ΔPM_2.5_) was calculated relative to the pre-pandemic baseline, enabling estimation of period-specific changes in attributable mortality linked to mobility restrictions and economic reactivation [23,68].

#### 2.6.5. Uncertainty Analysis

Uncertainty associated with the estimates was evaluated using three complementary approaches: (1) analytical error propagation incorporating the variance of the β coefficient, (2) Monte Carlo simulations (n = 10,000) with probability distributions assigned to β and baseline mortality rates, and (3) sensitivity analyses varying β across its 95% confidence interval bounds and using alternative sources (Burnett et al. [58], GBD 2019 study [60], Pope et al. [91], WHO Air Quality Guidelines [74]) [94,95]. Final confidence intervals reflect the combined uncertainty of all model parameters.

#### 2.6.6. Model Assumptions and Limitations

The health impact assessment relies on several key assumptions: (a) a log-linear, no-threshold relationship between PM_2.5_ and mortality, (b) applicability of β coefficients derived from international cohort studies to the local population, (c) uniform population exposure represented by ambient concentrations at the monitoring station, and (d) no major residual confounding from socioeconomic or behavioral factors not explicitly controlled [74,91]. These assumptions, common in environmental health impact assessments, may introduce exposure misclassification and residual confounding; their implications are considered in the interpretation of results. Recent studies have noted that lockdown-related PM_2.5_ changes in some regions did not translate to expected mortality reductions due to complex interactions between meteorology, secondary aerosol formation, and population exposure patterns [63].

### 2.7. Data Visualization

All figures were generated in R using ggplot2 [96]. Boxplots summarized pollutant distributions by pandemic phase with WHO 2021 guideline lines as references. Time series were plotted with LOESS smoothing and 95% confidence bands. Scatterplots showed pollutant–meteorology relationships, using viridis color scales for temperature coding [97]. Variance decomposition results were displayed as stacked bar charts, and percentage changes included 95% confidence intervals. Concentration–response curves were plotted following visualization approaches from global burden of disease assessments [51,58]. All graphics were exported at 600 dpi as TIFF (with embedded fonts) and PDF for publication-quality output.

### 2.8. Software and Reproducibility

All analyses were conducted in R version 4.5.1 [76] using RStudio as the integrated development environment. Data handling and visualization were performed with tidyverse [98] and related packages (lubridate for date manipulation [99], ggplot2 for graphics [96]). Random Forest models were implemented with the randomForest package [85]. Fully annotated R code for data preprocessing, statistical modeling, variance decomposition, and figure generation is provided as Supplementary File S1 to ensure reproducibility [87]. Statistical significance was set at α = 0.05, and effect sizes (e.g., *R*^2^) are reported alongside *p*-values where relevant [100].

## 3. Results

### 3.1. Air Pollutant Concentrations Across COVID-19 Pandemic Phases

Table 1 summarizes descriptive statistics for daily concentrations of PM_10_, PM_2.5_, NO_2_, O_3_, and SO_2_ across the six study phases. During the pre-pandemic baseline, median PM_10_ (48.1 µg/m^3^) already exceeded the WHO 2021 24 h guideline (45 µg/m^3^), while SO_2_ exhibited high variability (range: 7.9–129.9 µg/m^3^), reflecting episodic emissions from the copper smelter. Contrary to global trends of improved air quality during lockdowns, particulate matter concentrations increased during restriction phases. PM_2.5_ peaked in Phase 1 (16.9 ± 6.1 µg/m^3^), representing a 34% increase relative to pre-pandemic levels, before declining below baseline in Phase 3 (10.9 ± 3.4 µg/m^3^). PM_10_ showed similar patterns, with the highest median during Strict Lockdown (51.4 µg/m^3^) and the lowest in Phase 3 (33.6 µg/m^3^). In contrast, gaseous pollutants followed expected patterns of reduced concentrations. NO_2_ decreased consistently across all phases, ranging from 4.7 to 5.1 µg/m^3^ compared to the pre-pandemic mean of 5.8 µg/m^3^. SO_2_ showed the most pronounced decline, with Phase 4 concentrations (8.9 ± 0.8 µg/m^3^) representing a 65% reduction from baseline. O_3_ exhibited a marked mid-year increase, with Phase 3 concentrations (30.3 ± 5.5 µg/m^3^) more than doubling pre-pandemic levels.

Of note, SO_2_ and O_3_ data availability was substantially reduced during Phase 4 due to instrument maintenance issues: SO_2_ measurements were limited to nine consecutive days at the beginning of the phase (27 September–5 October 2020; n = 9), and O_3_ data were available for only 19 days. The very low variability in SO_2_ during this limited period (SD = 0.83 µg/m^3^) suggests consistently low emissions from the smelter complex; however, the reduced sample sizes should be considered when interpreting Phase 4 trends for these pollutants.

**Figure 3:**
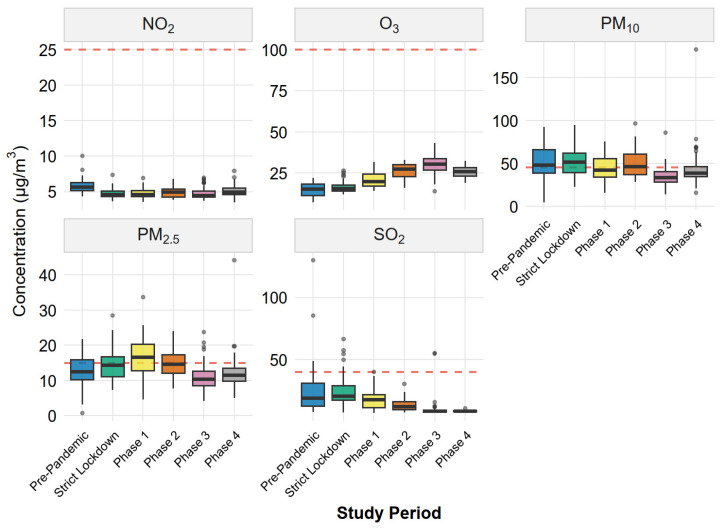
Daily air pollutant concentrations during six COVID-19 pandemic phases in Ilo, Peru. Boxplots show distributions of daily mean concentrations of PM_10_, PM_2.5_, NO_2_, O_3_, and SO_2_. Boxes represent interquartile ranges (IQRs), center lines indicate medians, whiskers extend to 1.5 × IQR, and points represent outliers. Red dashed horizontal lines mark WHO (2021) 24 h air quality guideline values. Phase definitions are provided in Table 1.

Figure 3 visualizes the distributional characteristics of pollutant concentrations across phases. PM_10_ distributions consistently exceeded the WHO 24 h guideline (45 µg/m^3^), with median values above the threshold in all phases except Phase 3. For PM_2.5_, the guideline (15 µg/m^3^) was exceeded primarily during Phase 1, which also exhibited the widest interquartile range and highest outliers, indicating substantial day-to-day variability during early reopening. NO_2_, O_3_, and SO_2_ remained below their respective WHO thresholds throughout the study period. Notably, SO_2_ showed progressively narrower distributions from pre-pandemic to Phase 4, with reduced outlier frequency, suggesting more consistent (and lower) emissions from the smelter complex during later phases. O_3_ displayed an opposite pattern, with an expanding IQR and increasing medians from Strict Lockdown through Phase 3, consistent with enhanced photochemical production during winter months.

**Figure 4:**
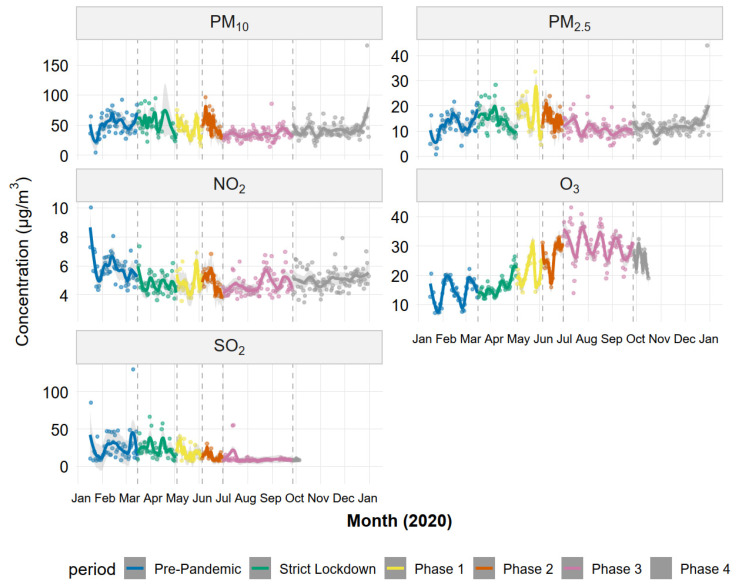
Temporal evolution of daily air pollutant concentrations during the COVID-19 pandemic in Ilo, Peru. Daily mean concentrations (points) and LOESS-smoothed trends (solid lines; span = 0.3) are shown for PM_10_, PM_2.5_, NO_2_, O_3_, and SO_2_ throughout 2020. Vertical dashed lines indicate boundaries between the six COVID-19 phases. Phase color coding is shown in the legend.

Figure 4 shows the temporal evolution of daily pollutant concentrations throughout 2020. LOESS-smoothed trends reveal distinct patterns for each pollutant class. PM_10_ and PM_2.5_ exhibited similar trajectories: relatively stable during pre-pandemic and Strict Lockdown, peaking during Phases 1–2 (May–June), and declining to annual minima during Phase 3 (July–September) before partial recovery in Phase 4. Gaseous pollutants displayed contrasting temporal dynamics. NO_2_ showed an immediate step-decrease at lockdown onset (mid-March) that persisted throughout the year, with day-to-day variability substantially reduced compared to the pre-pandemic period. SO_2_ exhibited the most dramatic temporal pattern: high and erratic concentrations during pre-pandemic (reflecting episodic smelter emissions), followed by progressive decline and stabilization at low levels from Phase 2 onward. O_3_ displayed a pronounced seasonal cycle, with concentrations increasing steadily from April through September (Phases 1–3) before declining in Phase 4, consistent with enhanced photochemical production during austral winter when reduced NO_x_ favored ozone accumulation.

**Figure 5:**
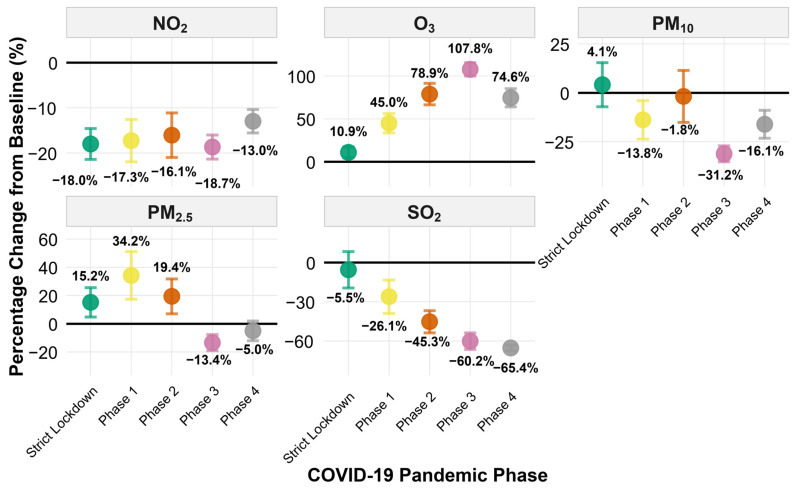
Percentage change in daily air pollutant concentrations relative to the pre-pandemic baseline during COVID-19 restriction phases in Ilo, Peru. Panels show estimated percentage changes in mean concentrations of NO_2_, O_3_, PM_10_, PM_2.5_, and SO_2_ for each restriction phase compared with the Pre-Pandemic period (1 January–15 March 2020). Points represent mean percentage changes, and vertical error bars denote 95% confidence intervals. The horizontal black line marks zero change. Numeric labels display percentage values for each phase.

Figure 5 quantifies percentage changes in pollutant concentrations relative to the pre-pandemic baseline. The five pollutants exhibited markedly different response patterns to COVID-19 restrictions. NO_2_ showed consistent reductions across all phases (−13% to −19%), with overlapping confidence intervals indicating sustained decreases regardless of restriction intensity. SO_2_ displayed the largest and most progressive decline, exceeding −60% by Phases 3–4, reflecting reduced smelter operations. In contrast, O_3_ increased substantially throughout the restriction period, peaking at +108% during Phase 3; this counterintuitive response is consistent with reduced NO titration in a NO_x_-saturated regime, where lower NO emissions allow ozone to accumulate. Particulate matter showed divergent patterns between phases. PM_2.5_ increased during Strict Lockdown (+15%) and Phase 1 (+34%), then declined below baseline in Phases 3–4 (−5% to −13%). PM_10_ exhibited greater variability, with modest increases during Strict Lockdown (+4%) and Phase 2 (−2%), but substantial reductions during Phase 3 (−31%). The wide confidence intervals for PM_10_ reflect high day-to-day variability influenced by meteorological conditions. Detailed percentage and absolute changes for all pollutants are provided in Supplementary Table S2.

### 3.2. Meteorological Conditions and Pollutant–Meteorology Relationships

**Figure 6:**
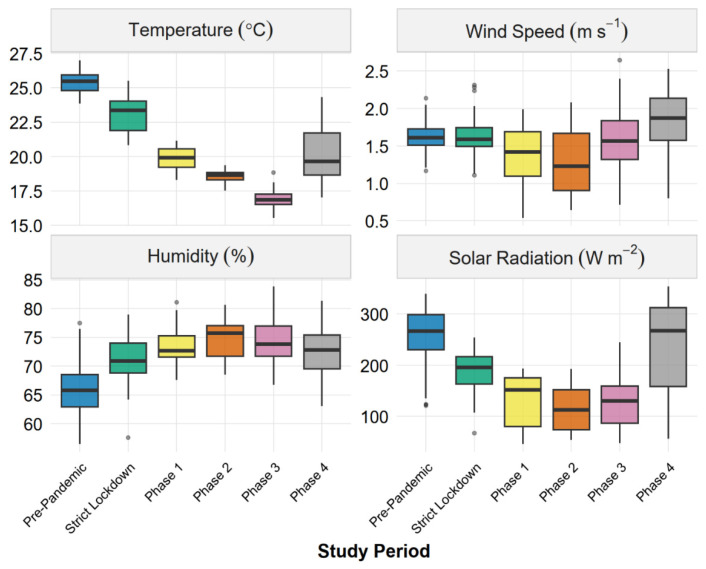
Daily meteorological conditions during six COVID-19 pandemic phases in Ilo, Peru. Boxplots show the distributions of daily mean temperature (°C), wind speed (m·s^−1^), relative humidity (%), and solar radiation (W·m^−2^) for each study phase. Boxplot elements represent interquartile range, median, whiskers to 1.5 × IQR, and outliers. These meteorological variables were included as continuous covariates in the multiple regression models (see Section 3.3).

Meteorological conditions varied systematically across phases, reflecting the austral seasonal transition from summer to winter (Figure 6). Temperature showed a clear declining trend from pre-pandemic (median ~25 °C) through Phase 3 (median ~17 °C), with Phase 3 exhibiting both the lowest values and narrowest distribution, before partial recovery in Phase 4. Relative humidity displayed an inverse pattern, increasing from pre-pandemic levels (~66%) to peak values during Phases 1–3 (>73%), consistent with the intensification of the coastal marine layer during cooler months. Solar radiation followed the expected seasonal cycle, with substantially reduced values and compressed distributions during Phases 1–3 (winter) compared to pre-pandemic and Phase 4 (summer). Wind speed showed less pronounced seasonality, though Phases 1–2 exhibited lower medians and occasional very low outliers that may have contributed to reduced pollutant dispersion during these periods. These systematic meteorological changes coincide temporally with the observed pollutant variations (Section 3.1), underscoring the need to account for weather-related confounding when attributing concentration changes to COVID-19 restrictions.

**Figure 7:**
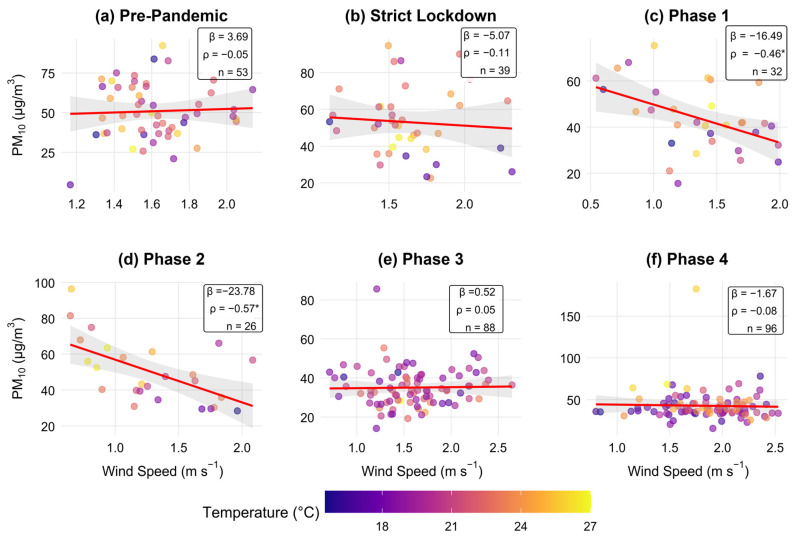
Phase-specific relationships between daily PM_10_ concentrations and wind speed during the COVID-19 pandemic in Ilo, Peru. Panels (**a**–**f**) show scatter plots for each study phase. Points represent daily mean values and are colored by temperature (°C) using a plasma palette (cooler days in purple, warmer days in yellow). Red lines indicate ordinary least-squares regression fits with 95% confidence intervals (grey shaded bands). Insets report the regression slope β (µg m^−3^ per m s^−1^), Spearman correlation coefficient ρ, and number of days n; asterisks denote statistically significant associations (*p* < 0.05).

Correlation analysis (Supplementary Figure S1) revealed coherent relationships among meteorological variables and pollutants. PM_10_ and PM_2.5_ were positively associated with temperature and negatively with wind speed, while O_3_ showed strong negative correlations with NO_2_ and humidity, consistent with photochemical suppression under cloudy conditions. These correlation structures supported the selection of temperature, wind speed, and humidity as covariates in subsequent regression models. Day-of-week patterns (Supplementary Figure S2) indicated that NO_2_ exhibited a pronounced weekday–weekend contrast during pre-pandemic and Strict Lockdown periods, which largely disappeared during Phases 2–4, suggesting sustained reductions in vehicular activity beyond the initial lockdown. Phase-specific relationships between PM_10_ and wind speed are shown in Figure 7. The wind–PM_10_ association was not temporally stationary: during Pre-Pandemic (β = 3.69, ns) and Phase 3 (β = 0.52, ns), relationships were weak and non-significant, whereas Phases 1–2 exhibited strong negative slopes (β = −16.5 to −23.8, *p* < 0.05), indicating enhanced dispersion effects. This phase-dependent heterogeneity coincided with temperature differences visible in the color gradient—cooler conditions (purple points) dominated Phases 1–3, while warmer days (yellow points) characterized Pre-Pandemic and Phase 4. The reversal of the wind–PM_10_ relationship underscores that meteorological effects on pollutant dispersion vary seasonally and cannot be assumed constant across the study period.

### 3.3. Relative Contributions of COVID-19 Restrictions and Meteorology

**Figure 8:**
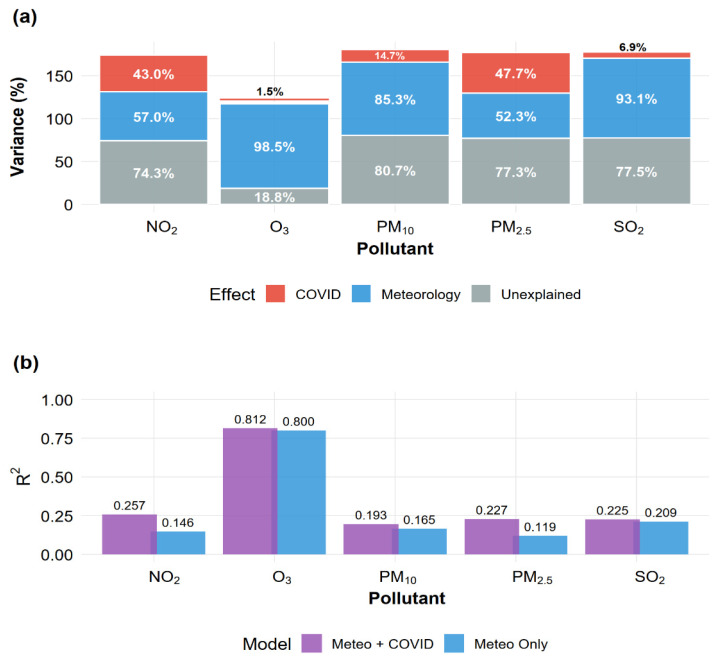
Relative contributions of meteorology and COVID-19 restriction periods to variability in daily air pollutant concentrations in Ilo, Peru. (**a**) Variance decomposition based on multiple linear regression models. Grey bars represent unexplained variance (100 × (1 − R^2^)); blue and red segments show fractions of explained variance attributable to meteorology and COVID-19 periods, respectively. Percentages within the explained component are labeled. (**b**) Model performance comparison showing R^2^ for meteorology-only models (light blue) and full models including COVID-19 phases (purple). Numeric labels indicate R^2^ values.

To disentangle meteorological from anthropogenic influences, we fitted nested multiple linear regression models: a meteorology-only model (temperature, wind speed, humidity) and a full model adding COVID-19 phase indicators. Variance decomposition and ANOVA results are summarized in Table 2 and visualized in Figure 8. The five pollutants exhibited distinct variance structures (Figure 8a). O_3_ was almost entirely meteorology-driven, with 98.5% of explained variance attributable to weather variables; the marginal COVID-19 contribution (1.5%) was statistically significant (*p* = 0.005) but negligible in magnitude.

This pattern reflects the strong coupling between ozone photochemistry and seasonal meteorological cycles. PM_2.5_ showed the most balanced partitioning (52% meteorology, 48% COVID-19), indicating that both weather conditions and restriction phases contributed substantially to observed variability. NO_2_ displayed a similar pattern, though slightly more meteorology-influenced (57% vs. 43%). PM_10_ was predominantly meteorology-controlled (85%), with restriction phases contributing only 15% of explained variance. For SO_2_, the full model explained only about 23% of the total variance, with the majority of the explained component attributed to meteorology. The non-significant incremental contribution of COVID-19 periods (*p* = 0.44) and the large unexplained variance suggest that episodic smelter-related emissions and unmeasured synoptic factors dominate SO_2_ variability beyond the resolution of our phase-based approach. This reflects high day-to-day variability driven by episodic smelter emissions not captured by either meteorological variables or phase definitions, resulting in limited statistical power despite large observed concentration changes. Model performance varied considerably across pollutants (Figure 8b). The full model explained 81% of O_3_ variance but only 19–26% for other pollutants, indicating substantial unexplained variability likely driven by factors not included in the models (e.g., boundary layer height, synoptic patterns, source-specific emissions). ANOVA F-tests (Table 2) confirmed that COVID-19 phases added significant explanatory power beyond meteorology for four of five pollutants (PM_10_: *p* = 0.045; PM_2.5_: *p* = 0.001; NO_2_: *p* = 0.001; O_3_: *p* = 0.005), with SO_2_ being the exception. Full regression coefficients are provided in Supplementary Table S3; notably, all phases showed negative NO_2_ coefficients (sustained reductions) and positive PM_2.5_ coefficients (elevated concentrations relative to baseline despite lockdowns). Random Forest models provided a complementary non-parametric assessment (Supplementary Figure S3, Table S4). Variable importance rankings were broadly consistent with regression-based decomposition: meteorological variables dominated for O_3_ (>60% combined importance), while COVID-19 period and meteorology contributed comparably for PM_2.5_ and NO_2_. This agreement between parametric and non-parametric approaches supports the robustness of our variance partitioning conclusions (Supplementary Table S6 provides a detailed side-by-side comparison of regression-based variance fractions and Random Forest importance rankings). Model fit and diagnostic statistics are summarized in Supplementary Table S5, and residual diagnostics (Supplementary Figure S4) confirmed that model assumptions were reasonably satisfied for all pollutants.

### 3.4. Health Impact Assessment of PM_2.5_ Changes

**Figure 9:**
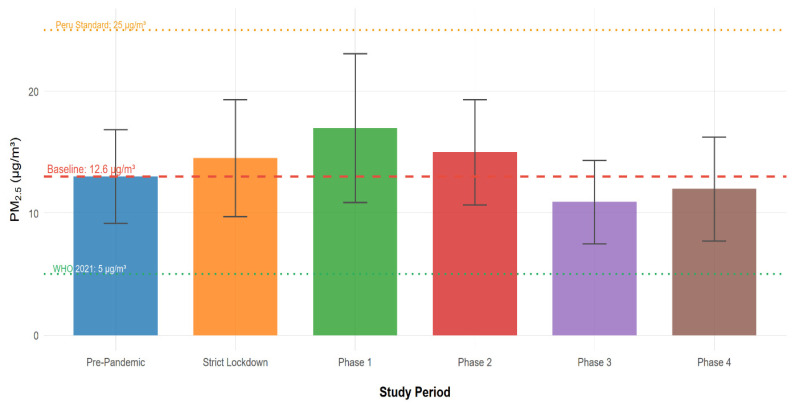
Average PM_2.5_ concentrations by COVID-19 restriction phase in Ilo, Peru (2020). Bars represent mean daily PM_2.5_ concentrations (µg/m^3^) with error bars indicating ± 1 SD. Red dashed line: Pre-Pandemic baseline (12.60 µg/m^3^). Green dotted line: WHO 2021 annual guideline (5 µg/m^3^). Yellow dotted line: Peruvian national standard (25 µg/m^3^).

Given its well-established cardiovascular effects and the availability of robust concentration–response functions, PM_2.5_ was selected for health impact assessment. Figure 9 displays phase-specific PM_2.5_ concentrations, while Table 3 presents the corresponding relative risks and attributable mortality estimates for the population of Ilo.

The Pre-Pandemic mean PM_2.5_ concentration (12.6 ± 4.36 µg/m^3^) served as the reference baseline. This value already exceeded the WHO 2021 annual guideline (5 µg/m^3^) by more than two-fold, indicating that the population was exposed to health-relevant PM_2.5_ levels even before the pandemic. During the restriction phases, PM_2.5_ followed a biphasic pattern: concentrations increased during Strict Lockdown and Phases 1–2, peaking in Phase 1 (16.91 ± 6.14 µg/m^3^), before declining below baseline during Phases 3–4 (Figure 9). Notably, even the lowest phase mean (Phase 3: 10.91 µg/m^3^) remained above the WHO guideline, underscoring persistent exposure to potentially harmful PM_2.5_ levels throughout the study period.

Applying the GBD 2021 concentration–response coefficient (β = 0.00072 per 1 µg/m^3^), these concentration changes translated into quantifiable mortality impacts (Table 3). The elevated PM_2.5_ during Phase 1 yielded the highest relative risk (RR = 1.003) and an estimated 0.23 additional cardiovascular deaths per year—the largest attributable burden observed. The position of phase-specific PM_2.5_ concentrations along the integrated exposure–response curve (Supplementary Figure S5) illustrates how relatively small changes in the 10–20 µg/m^3^ range translate into measurable differences in cardiovascular risk.

Strict Lockdown and Phase 2 produced smaller but still positive attributable mortality (0.09 and 0.11 deaths, respectively). Conversely, the reduced PM_2.5_ concentrations during Phases 3 and 4 were associated with avoided mortality (−0.12 and −0.06 deaths, respectively), representing a protective effect relative to baseline conditions.

Although these absolute numbers appear small for a city of ~67,000 inhabitants, they reflect the epidemiological reality that PM_2.5_ health effects operate across entire populations and accumulate over time. When extrapolated to larger urban areas with similar exposure patterns, such increments could translate into dozens to hundreds of attributable deaths annually. Moreover, the confidence intervals around our estimates (Table 3) indicate that the true mortality burden during Phase 1 could have ranged from 0.15 to 0.34 additional deaths per year, representing meaningful uncertainty that should inform precautionary approaches to air quality management.

The net health impact across the full pandemic period was approximately neutral: the additional mortality attributable to elevated PM_2.5_ during early restriction phases was largely offset by avoided deaths during later phases when concentrations declined. However, this aggregate balance masks the temporal heterogeneity of health burdens and underscores that meteorologically driven PM_2.5_ increases, even during periods of reduced anthropogenic emissions, can generate measurable public health consequences.

## 4. Discussion

### 4.1. Air Quality Responses to COVID-19 Measures in a Coastal Latin American City

The multi-pollutant response observed in Ilo only partially fits the global narrative of “cleaner air” during COVID-19 lockdowns. Most multi-city syntheses report sharp and widespread reductions in NO_2_ (about 20–60%) and more modest, heterogeneous changes in particulate matter, with small decreases or even increases depending on local sources and meteorology [10,21,37,101]. In Ilo, we also detected persistent NO_2_ reductions and substantial SO_2_ declines consistent with traffic and industrial curtailment. Daily mean NO_2_ decreased by 13–19% across all pandemic phases relative to the pre-pandemic baseline, while SO_2_ concentrations fell by roughly 65% by Phase 4, with a marked tightening of the distribution and disappearance of high-emission episodes. In contrast, both PM_10_ and PM_2.5_ increased during the early restriction phases—most notably during Phase 1, when PM_2.5_ rose from 12.6 ± 4.4 µg/m^3^ in the Pre-Pandemic period to 16.9 ± 6.1 µg/m^3^ (+34%) and PM_10_ remained above the WHO 24 h guideline in most days—despite reduced mobility and industrial activity. Similar counterintuitive PM responses have been reported in some European and Asian cities once meteorological confounding is accounted for, with increases attributed to changes in boundary-layer height, secondary formation, and non-traffic sources rather than to policy failure [12,24,38,101]. Our findings therefore reinforce that lockdowns acted as a “natural experiment” in which emissions, atmospheric chemistry, and meteorology interacted in complex, context-specific ways rather than producing uniform improvements.

The magnitude and persistence of NO_2_ reductions in Ilo are comparable to those reported for other traffic-dominated urban areas, where mobility restrictions, teleworking, and behavioral changes produced large and sustained decreases in near-roadway NO_2_ even beyond strict lockdown periods [21,37,101,102]. The progressive SO_2_ decline, by contrast, is more typical of settings dominated by a small number of industrial point sources, where operational decisions at smelters or power plants drive most temporal variability [103]. Together, these patterns place Ilo within the broader Latin American experience, where studies in megacities such as Mexico City, São Paulo, and Lima have documented sharp NO_2_ decreases, more moderate and spatially heterogeneous PM responses, and strong contrasts linked to local source mixes and meteorological regimes [43,44,102,103,104,105]. In our case, the combination of increased PM_2.5_ during early phases and large mid-year ozone increases (+108% in Phase 3 relative to Pre-Pandemic) underscores how coastal meteorology and photochemistry can partially offset or even reverse the air-quality benefits of short-term emission reductions.

The counterintuitive PM_2.5_ increases during lockdown phases in Ilo can be attributed to several interconnected meteorological and geographic mechanisms characteristic of coastal industrial cities. First, the lockdown period coincided with the transition from austral summer to winter (March–July), characterized by reduced solar radiation, lower temperatures, and decreased boundary layer heights. These conditions promote atmospheric stability and reduce vertical mixing, allowing pollutants to accumulate near the surface regardless of emission reductions. Our variance decomposition results showing temperature as the dominant meteorological predictor for PM_2.5_ support this interpretation.

Second, Ilo’s coastal location introduces complex sea-breeze dynamics. During winter months, the land–sea thermal gradient weakens, reducing the ventilation effect of onshore winds that typically disperse pollutants during summer. The significant negative relationship between wind speed and PM_2.5_ concentrations in our regression models (Table S3) confirms that reduced wind speeds contributed to elevated concentrations during lockdown.

Third, while traffic-related emissions decreased during restrictions, the Southern Peru Copper Corporation (SPCC) smelter the city’s major stationary source maintained continuous operations as an essential industry. This created a scenario where the meteorological suppression of dispersion outweighed the modest emission reductions from reduced vehicular activity. The chemical speciation data showing elevated Cu, Pb, and As concentrations during lockdown phases (particularly Phase 2) further supports the dominant influence of industrial sources.

Finally, secondary aerosol formation may have been enhanced during the cold, humid winter months. The moderate positive relationship between humidity and PM_2.5_ in our models suggests that hygroscopic growth and aqueous-phase chemistry contributed to particle mass accumulation, particularly for sulfate aerosols derived from SO_2_ emissions. These findings align with observations from other coastal industrial cities globally, where local meteorological controls can override emission reductions during lockdowns [12,24,106,107].

### 4.2. Disentangling Restriction Measures and Meteorology: Pollutant-Specific Patterns

A central contribution of this work is the explicit variance decomposition of meteorological versus COVID-19 period effects. The nested regression results show that PM_2.5_ and NO_2_ are controlled by a balance between emission changes and meteorology, whereas O_3_ is overwhelmingly meteorologically driven and SO_2_ is dominated by anthropogenic changes linked to smelter operation. Specifically, for PM_2.5_, the full model explained about one quarter of the total variance (R^2^ ≈ 0.23), with approximately 52% of the explained variance attributable to meteorology and 48% to COVID-19 phases. For NO_2_, the full model explained roughly 25.7% of variance, partitioned into ≈57% meteorological and 43% COVID-19 contributions. PM_10_ was predominantly meteorology-controlled, with around 85% of explained variance attributable to weather and only 15% to restriction phases, consistent with a strong contribution from natural coarse-mode sources.

Ozone exhibited a different behavior: the full model accounted for 81.2% of O_3_ variance, of which 98.5% was explained by meteorology and only 1.5% by COVID-19 phases. Although this marginal COVID-19 contribution was statistically significant (*p* = 0.005), its magnitude was negligible compared with the dominant seasonal and meteorological controls. This hierarchy is consistent with process-based understanding: ozone production and loss are tightly coupled to temperature, solar radiation, humidity, and mixing depth, while NO_2_ responds rapidly to changes in traffic and atmospheric dispersion; PM_2.5_ reflects a mixture of primary and secondary sources with both natural and anthropogenic components [24,38,42,101].

The strong meteorological control on O_3_, combined with only a small but significant COVID-19 contribution, mirrors findings from regional and global analyses showing that ozone responses to lockdowns were modest and often opposite in sign to NO_2_, with increases linked to reduced NO titration in VOC-limited regimes [21,37,38,101]. In Ilo, the U-shaped seasonal pattern higher O_3_ in winter despite lower solar radiation—together with strong negative O_3_–NO_2_ correlations—suggests a NO_x_-saturated coastal environment where reductions in NO_x_ emissions favor ozone accumulation, particularly under stable, humid conditions. This behavior underscores that NO_x_ control strategies may increase O_3_ in some coastal or port cities unless accompanied by VOC reductions, a phenomenon widely discussed in the COVID-19 literature [37,38,42].

For PM_2.5_, the nearly balanced partition between meteorology and COVID-19 periods is noteworthy. It implies that emission changes associated with mobility and industrial restrictions were large enough to be detectable even after controlling for seasonal meteorology, but that unfavorable conditions during austral autumn–winter (cooler temperatures, weaker winds, higher humidity) offset part of these benefits. The phase-dependent wind–PM_10_ relationships, with strong negative slopes during Phases 1–2 and weak or non-significant associations in Pre-Pandemic and Phase 3, further highlight that dispersion effects varied across the year and cannot be assumed stationary. Similar behavior has been reported in other coastal or port-influenced cities, where sea-salt aerosols, crustal dust, and secondary formation contribute importantly to PM loadings and are strongly modulated by boundary-layer dynamics [12,24,55,106,107].

The Random Forest analysis provides an important robustness check. Despite relaxing linearity assumptions and allowing for interactions, the non-parametric models yielded variable-importance rankings broadly consistent with the regression-based variance decomposition: meteorological variables dominated for O_3_ (with temperature and humidity jointly accounting for more than 60% of RF importance), while meteorology and COVID-19 phase had comparable importance for PM_2.5_ and NO_2_. COVID-19 period remained a key predictor across pollutants, even when non-linear effects were allowed. Together, these complementary approaches support the conclusion that, in Ilo, the air-quality impacts of COVID-19 restrictions cannot be interpreted without explicit consideration of meteorological variability [34,35,36,54,75].

### 4.3. Health Relevance and Implications for Air Quality Management

The health impact assessment translates the modest concentration changes observed for PM_2.5_ into concrete public-health metrics. Using concentration–response functions from the Global Burden of Disease framework [2] and large cohort studies, long-term exposure to PM_2.5_ in the 10–20 µg/m^3^ range typical of Ilo is associated with small but non-negligible increases in cardiovascular mortality risk [1,58,62,108]. In our data, the pre-pandemic mean PM_2.5_ concentration (12.6 ± 4.4 µg/m^3^) already exceeded the WHO 2021 annual guideline (5 µg/m^3^) by more than a factor of two, indicating that the population was exposed to health-relevant levels even before COVID-19.

During the restriction phases, PM_2.5_ followed a biphasic pattern: concentrations increased during Strict Lockdown and Phase 1, peaking in Phase 1 (16.9 ± 6.1 µg/m^3^), before declining below baseline during Phases 3 (10.9 ± 3.4 µg/m^3^) and 4 (12.0 ± 4.4 µg/m^3^). Applying the GBD 2021 coefficient (β = 0.00072 per 1 µg/m^3^), these concentration changes translated into an estimated 0.23 additional cardiovascular deaths per year during Phase 1 and smaller positive burdens during Strict Lockdown (0.09) and Phase 2 (0.11). Conversely, reduced PM_2.5_ concentrations during Phases 3 and 4 were associated with avoided deaths (−0.12 and −0.06 per year, respectively). While these absolute numbers are modest for a city of about 67,000 inhabitants, they illustrate that even apparently small concentration shifts of 3–5 µg/m^3^ can yield detectable changes in attributable mortality when applied to entire populations [62,63,90,108]. Scaling these patterns to larger coastal urban corridors would translate into tens to hundreds of additional or avoided deaths annually.

These findings reinforce three key points for air-quality and health policy. First, meteorologically driven increases in PM_2.5_ during periods of emission reduction can still generate measurable health burdens; evaluating interventions based solely on emissions or raw concentrations without meteorological normalization risks misinterpretation, including underestimation of risks associated with “unfavorable” weather. Second, the coexistence of clear benefits for gaseous pollutants (NO_2_, SO_2_) with ambiguous or adverse responses for PM underscores the need for multi-pollutant, source-specific strategies rather than single-pollutant approaches. In Ilo, complementary control of industrial SO_2_, traffic-related NO_2_, and sources contributing to PM_2.5_ (including secondary formation and marine-influenced components) will be needed to achieve meaningful health benefits. Third, the fact that all pandemic phases remained well above the WHO 2021 guideline for annual PM_2.5_, even in the “cleanest” period, underscores that substantial additional emission reductions—beyond those induced by temporary lockdowns—are required to align with health-protective targets [58,62,74,108].

### 4.4. Strengths, Limitations, and Future Directions

This study has several strengths. Methodologically, the combination of nested regression models, variance decomposition, and machine learning variable-importance analysis provides a transparent and robust framework to quantify the relative roles of meteorology and policy interventions, complementing more traditional “before–after” comparisons that may be biased by weather conditions [12,24,34,35,36,101]. Substantively, the work adds evidence from a coastal, industrial Latin American city—a setting under-represented in the COVID-19 air-quality literature relative to Asia, Europe, and North America [102,104,108,109]. To our knowledge, this is one of the first studies in a medium-sized Latin American coastal industrial city that jointly (i) decomposes meteorological and COVID-19 phase contributions to multi-pollutant variability using both regression-based and Random Forest approaches and (ii) translates phase-specific PM_2.5_ changes into cardiovascular mortality estimates. This dual focus on mechanistic drivers and health implications enhances the policy relevance of the findings.

At the same time, important limitations should be acknowledged. First, the analysis relies on a single urban monitoring site located at the National University of Moquegua, which may not capture intra-urban gradients near major roads, the port, or the smelter complex. Given that our health impact assessment assumes homogeneous population exposure, this spatial limitation warrants consideration. However, several factors support the representativeness of this site for Ilo’s population (~67,000 inhabitants): (i) the city’s compact urban area (~15 km^2^) and relatively flat coastal topography favor well-mixed atmospheric conditions; (ii) the monitoring station is situated in an open area free from local emission sources, representing background urban concentrations; and (iii) the dominant sea-breeze circulation promotes relatively uniform pollutant dispersion across the urban core [106,107]. Nevertheless, residents living near the smelter or port facilities may experience higher exposures than those captured by this central monitoring site. Future work combining fixed monitoring, mobile campaigns, and low-cost sensor networks could resolve spatial contrasts and identify micro-environments with disproportionate health risks [110]. Second, the meteorological characterization is constrained to standard surface variables; explicit treatment of boundary-layer height, synoptic circulation, and marine stratocumulus regimes using radiosonde, ceilometer, or reanalysis data would refine attribution of seasonal patterns, particularly for ozone and PM [24,42,106]. Third, the health-impact assessment focuses on cardiovascular mortality and long-term concentration–response functions applied to relatively short phases; broader outcome spectra (respiratory mortality, hospital admissions, morbidity) and alternative short-term coefficients would provide a more complete picture of health burden [1,62,94,111].

Finally, the observational nature of the COVID-19 “experiment” limits causal inference: restrictions co-occurred with behavioral, economic, and climatic changes that cannot be fully disentangled. Integrating these empirical findings with source-apportionment (e.g., receptor models, chemical speciation), chemical-transport modeling, and remote sensing would allow more mechanistic attribution of pollutant responses to specific source categories and processes. Such work is particularly relevant for coastal industrial cities like Ilo, where port activities, shipping, smelting, traffic, and natural marine aerosols interact within a narrow coastal boundary layer [55,106,107,112].

Taken together, our results highlight that (i) meteorological normalization is indispensable for evaluating air-quality interventions, (ii) PM_2.5_, NO_2_, and SO_2_ respond differently to the same policy shock because of their distinct source and process controls, and (iii) even modest concentration changes around relatively low baselines can have non-trivial health implications. For Peru and other coastal cities in the global South, these lessons suggest that temporary mobility restrictions are insufficient to approach WHO PM_2.5_ guidelines; instead, sustained structural measures targeting industrial point sources, port emissions, traffic, and regional background pollution designed and evaluated within a meteorologically normalized, multi-pollutant framework will be required to achieve durable public-health benefits [4,105,109].

## 5. Conclusions

This study analyzed air-quality responses to COVID-19 restrictions in Ilo, a medium-sized coastal industrial city in southern Peru, a setting underrepresented in global COVID-19 air-pollution research. Using multiple linear regression, variance decomposition, Random Forest models and a health impact assessment, we quantified how meteorology modulated the effects of mobility restrictions and industrial shutdowns on PM_10_, PM_2.5_, NO_2_, O_3_ and SO_2_ during six pandemic phases in 2020. Our results show pollutant-specific responses that challenge the notion of uniformly improved air quality during lockdowns. NO_2_ decreased consistently by 13–19% and SO_2_ by up to 65% under reduced smelter activity, yet PM_2.5_ increased by 34% during the early reopening phase (Phase 1) despite substantial reductions in traffic and industry. O_3_ concentrations more than doubled (+108%) in austral winter, reflecting NO_x_-saturated coastal photochemistry. Variance decomposition indicated that O_3_ was almost entirely meteorology-driven (≈98% of explained variance), whereas PM_2.5_ and NO_2_ were jointly controlled by meteorology and COVID-19 phases (≈50% each), and SO_2_ remained predominantly influenced by smelter emissions. Random Forest results were consistent with these control hierarchies. Translating PM_2.5_ changes into health metrics using Global Burden of Disease functions showed that a ≈4 µg/m^3^ increase in Phase 1 produced a small but measurable excess in cardiovascular mortality, while later decreases were associated with avoided deaths. However, PM_2.5_ levels in all phases remained above WHO 2021 guideline values, indicating that larger and more sustained emission reductions are needed to meet health-protective targets. Overall, our findings highlight that meteorological normalization, multi-pollutant strategies and coastal-specific management approaches are essential for designing and evaluating effective emission-control policies in Peru and other coastal cities of the global South.

## Figures and Tables

**Table 1:** Descriptive statistics of daily air pollutant concentrations by COVID-19 pandemic phase in Ilo, Peru.

Pollutant	Period	M ± SD	Mdn	Range	n
PM_10_	Pre-Pandemic	50.93 ± 17.38	48.11	4.56–92.28	53
PM_10_	Strict Lockdown	53.04 ± 18.31	51.43	22.57–94.73	39
PM_10_	Phase 1	43.90 ± 14.41	41.79	15.66–75.32	32
PM_10_	Phase 2	50.00 ± 17.60	46.33	28.40–96.36	26
PM_10_	Phase 3	35.07 ± 9.90	33.63	14.18–85.62	88
PM_10_	Phase 4	42.74 ± 18.18	38.43	15.74–182.48	96
PM_2.5_	Pre-Pandemic	12.60 ± 4.36	12.39	0.71–21.71	53
PM_2.5_	Strict Lockdown	14.51 ± 4.70	14.23	7.33–28.42	49
PM_2.5_	Phase 1	16.91 ± 6.14	16.57	4.58–33.65	32
PM_2.5_	Phase 2	15.04 ± 4.06	14.61	7.74–23.96	26
PM_2.5_	Phase 3	10.91 ± 3.44	10.31	4.19–23.72	88
PM_2.5_	Phase 4	11.96 ± 4.40	11.48	5.08–44.13	96
NO_2_	Pre-Pandemic	5.81 ± 1.00	5.67	4.31–10.02	56
NO_2_	Strict Lockdown	4.77 ± 0.71	4.61	3.64–7.34	49
NO_2_	Phase 1	4.81 ± 0.78	4.56	3.58–6.91	32
NO_2_	Phase 2	4.88 ± 0.74	4.90	3.86–6.82	26
NO_2_	Phase 3	4.73 ± 0.74	4.47	3.72–6.96	88
NO_2_	Phase 4	5.06 ± 0.75	4.93	3.49–7.91	96
O_3_	Pre-Pandemic	14.59 ± 4.23	15.31	7.21–22.19	56
O_3_	Strict Lockdown	16.18 ± 3.38	15.44	12.03–26.45	49
O_3_	Phase 1	21.15 ± 4.79	19.70	14.23–31.69	32
O_3_	Phase 2	26.09 ± 4.75	27.25	15.98–33.09	26
O_3_	Phase 3	30.31 ± 5.47	30.37	13.96–43.20	88
O_3_	Phase 4	25.47 ± 3.56	25.80	19.02–32.40	19
SO_2_	Pre-Pandemic	25.57 ± 20.59	19.05	7.91–129.88	56
SO_2_	Strict Lockdown	24.17 ± 12.74	20.67	7.71–66.55	49
SO_2_	Phase 1	18.90 ± 9.46	17.78	7.24–40.18	32
SO_2_	Phase 2	14.00 ± 5.64	12.41	7.68–30.54	26
SO_2_	Phase 3	10.18 ± 7.41	8.83	7.10–55.30	79
SO_2_	Phase 4	8.85 ± 0.83	8.73	8.17–10.91	9

Note. M = mean; SD = standard deviation; Mdn = median; n = number of daily observations. All concentrations in µg/m^3^. Study phases: Pre-Pandemic (1 January–15 March 2020), Strict Lockdown (16 March–3 May), Phase 1 (4 May–4 June), Phase 2 (5–30 June), Phase 3 (1 July–26 September), Phase 4 (27 September–31 December). WHO 2021 24 h guideline values: PM_10_ = 45 µg/m^3^, PM_2.5_= 15 µg/m^3^, NO_2_ = 25 µg/m^3^, O_3_ = 100 µg/m^3^ (8 h), SO_2_ = 40 µg/m^3^. SO_2_ and O_3_ sample sizes in Phase 4 reflect reduced data availability due to instrument maintenance (SO_2_: 27 September–5 October only; O_3_: 19 days).

**Table 2:** Relative contributions of meteorological variability and COVID-19 restriction periods to variance in daily air pollutant concentrations in Ilo, Peru.

Pollutant	n	R^2^	R^2^ Meteo	R^2^ COVID	% Meteo	% COVID	F (5, df_2_)	*p*
PM_10_	334	0.193	0.165	0.029	85.3	14.7	2.30	0.045
PM_2.5_	344	0.227	0.119	0.108	52.3	47.7	9.40	0.001
NO_2_	347	0.257	0.146	0.110	57.0	43.0	10.02	0.001
O_3_	271	0.812	0.800	0.013	98.5	1.5	3.49	0.005
SO_2_	251	0.225	0.209	0.015	93.1	6.9	0.96	0.440

Note. n = number of valid daily observations; R^2^ = coefficient of determination for full model; R^2^ Meteo = variance explained by meteorological variables only (temperature, wind speed, relative humidity); R^2^ COVID = incremental variance explained by COVID-19 period indicators beyond meteorology; % Meteo and % COVID = relative percentage contributions to total explained variance (R^2^); F(5, df_2_) = F statistic from ANOVA comparing nested models, where 5 = degrees of freedom for COVID-19 phases and df_2_ = residual degrees of freedom; *p* = probability value. Reference category: Pre-Pandemic period (1 January–15 March 2020). Significant effects (*p* < 0.05) indicate that COVID-19 periods explain additional variance beyond meteorology.

**Table 3:** Health impact assessment results by COVID-19 phase in Ilo, Peru (2020).

Period	PM_2.5_ (μg/m^3^)	Δ PM_2.5_ (µg/m^3^)	% Change	RR (95% CI)	Deaths (95% CI)
Pre-Pandemic	12.60 ± 4.36	0	0	1.000 (1.000–1.000)	0.00 (0.00–0.00)
Strict Lockdown	14.51 ± 4.70	1.91	15.2	1.001 (1.001–1.001)	0.09 (0.06–0.13)
Phase 1	16.91 ± 6.14	4.31	34.2	1.003 (1.002–1.003)	0.23 (0.15–0.34)
Phase 2	15.04 ± 4.06	2.45	19.4	1.001 (1.001–1.002)	0.11 (0.08–0.17)
Phase 3	10.91 ± 3.44	−1.69	−13.4	0.998 (0.999–0.998)	−0.12 (−0.08–−0.18)
Phase 4	11.96 ± 4.40	−0.63	−5.0	0.999 (0.999–0.999)	−0.06 (−0.04–−0.09)

Note. PM_2.5_ values expressed as mean ± SD (µg/m^3^). Δ PM_2.5_ = absolute change relative to Pre-Pandemic baseline (µg/m^3^). % Change = percentage change in concentration relative to Pre-Pandemic. RR = Relative Risk calculated using concentration-response coefficient β = 0.00072 per 1 µg/m^3^ from GBD 2021. Deaths = estimated annual attributable cardiovascular deaths (95% CI). Positive values indicate additional mortality; negative values indicate avoided deaths. Population: 67,167 inhabitants (total population; GBD methodology); baseline cardiovascular mortality rate: 120 per 100,000 inhabitants/year.

## Data Availability

The air quality and meteorological datasets analyzed in this study were obtained from the environmental monitoring network operated by the Universidad Nacional de Moquegua (UNAM) in Ilo and are not publicly available due to institutional data-sharing agreements, but are available from the corresponding author on reasonable request. Population and cause-specific mortality data were derived from national statistics and the Global Burden of Disease 2021 study, which are publicly accessible from the respective data providers.

## Supplementary Materials

The following supporting information can be downloaded at: https://www.mdpi.com/article/10.3390/ijerph23020191/s1, Figure S1. Correlation matrix between daily air pollutant concentrations and meteorological variables in Ilo, Peru. Figure S2. Daily mean concentrations of PM_10_, PM_2.5_, NO_2_, O_3_ and SO_2_ are shown by day of week during the COVID-19 pandemic in Ilo, Peru. Figure S3. Relative importance of COVID-19 period and meteorological predictors for daily air pollutant concentrations in Ilo, Peru. Figure S4. Diagnostic plots for the multiple linear regression model of daily PM_10_ concentrations in Ilo, Peru. Figure S5. Concentration–response relationship between long-term PM_2.5_ exposure and cardiovascular mortality. Table S1. Technical specifications of equipment used for monitoring criteria air pollutants in Ilo, Peru, prior to the COVID-19 pandemic. Table S2. Detailed percentage and absolute changes in daily air pollutant concentrations relative to the pre-pandemic baseline. Table S3. Multiple linear regression coefficients for daily air pollutant concentration models in Ilo, Peru. Table S4. Variable importance from Random Forest models for daily air pollutant concentrations. Table S5. Model fit and diagnostic statistics for full multiple linear regression models (meteorology + COVID-19 periods). Table S6. Comparison of variance partitioning between multiple linear regression and Random Forest ap-proaches for daily air pollutant concentrations in Ilo, Peru.
