# Supplementary material for: Counterintuitive PM2.5 Increases During COVID-19 Lockdown in Ilo, Peru: Coastal Meteorology and Cardiovascular Implications
Source: Int J Environ Res Public Health. 2026 Jan 31;23(2):191. doi: 10.3390/ijerph23020191 (PMC12940600; doi:10.3390/ijerph23020191)
Supplement: Supplementary file 1 [file ijerph-23-00191-s001.zip › ijerph-4050026-supplementary.pdf]

Supplementary Materials

Table S1. Technical specifications of equipment used for monitoring criteria air pollutants in Ilo, Peru, prior to the COVID-19 pandemic.

Parameter	Equipment	Brand	Model	Flow (L/min)	Analysis method
$PM_{2.5}$<br>PM_{10}	Continuous Measurement Monitor	Thermo ScientificTM	5028i	16.67	Beta ray attenuation
SO_2	Automatic analyzer	Thermo ScientificTM	43i	0.5	Ultraviolet fluorescence
NO_2	Automatic analyzer	Thermo ScientificTM	42i	0.6 to 0.8	Chemiluminescence
O_3	Automatic analyzer	Thermo ScientificTM	49i	1 to 3	Ultraviolet absorption photometry

Table S2. Detailed percentage and absolute changes in daily air pollutant concentrations relative to the pre-pandemic baseline.

Pollutant	Period	M (SD)	Δ%	Δ	n
PM_{10}	Strict Lockdown	53.04 (18.31)	4.1%	+2.10	39
PM_{10}	Phase 1	43.90 (14.41)	−13.8%	−7.03	32
PM_{10}	Phase 2	50.00 (17.60)	−1.8%	−0.93	26
PM_{10}	Phase 3	35.07 (9.90)	−31.2%	−15.87	88
PM_{10}	Phase 4	42.74 (18.18)	−16.1%	−8.19	96
$PM_{2.5}$	Strict Lockdown	14.51 (4.70)	15.2%	+1.91	49
$PM_{2.5}$	Phase 1	16.91 (6.14)	34.2%	+4.31	32
$PM_{2.5}$	Phase 2	15.04 (4.06)	19.4%	+2.45	26
$PM_{2.5}$	Phase 3	10.91 (3.44)	−13.4%	−1.69	88
$PM_{2.5}$	Phase 4	11.96 (4.40)	−5.0%	−0.63	96
NO_2	Strict Lockdown	4.77 (0.71)	−18.0%	−1.05	49
NO_2	Phase 1	4.81 (0.78)	−17.3%	−1.00	32
NO_2	Phase 2	4.88 (0.74)	−16.1%	−0.93	26
NO_2	Phase 3	4.73 (0.74)	−18.7%	−1.09	88
NO_2	Phase 4	5.06 (0.75)	−13.0%	−0.75	96
O_3	Strict Lockdown	16.18 (3.38)	10.9%	+1.59	49

Pollutant	Period	M (SD)	Δ%	Δ	n
O_3	Phase 1	21.15 (4.79)	45.0%	+6.56	32
O_3	Phase 2	26.09 (4.75)	78.9%	+11.51	26
O_3	Phase 3	30.31 (5.47)	107.8%	+15.72	88
O_3	Phase 4	25.47 (3.56)	74.6%	+10.89	20
SO_2	Strict Lockdown	24.17 (12.74)	−5.5%	−1.40	49
SO_2	Phase 1	18.90 (9.46)	−26.1%	−6.67	32
SO_2	Phase 2	14.00 (5.64)	−45.3%	−11.58	26
SO_2	Phase 3	10.18 (7.41)	−60.2%	−15.40	79
SO_2	Phase 4	8.85 (0.83)	−65.4%	−16.72	9

Note. M = mean concentration; SD = standard deviation; Δ% = percentage change relative to Pre-Pandemic period (January 1 – March 15, 2020); Δ = absolute change in $\mu g/m^3$ (positive values indicate increases, negative values indicate decreases); n = number of daily observations. Pre-Pandemic baseline concentrations: PM_{10} = 50.93 $\mu g/m^3$; $PM_{2.5}$ = 12.60 $\mu g/m^3$; NO_2 = 5.81 $\mu g/m^3$; O_3 = 14.59 $\mu g/m^3$; SO_2 = 25.57 $\mu g/m^3$. Changes reflect combined effects of COVID-19 restrictions and meteorological variability; see Table 2 for variance decomposition.

Table S3. Multiple linear regression coefficients for daily air pollutant concentration models in Ilo, Peru.

Pollutant	Predictor	B	SE	t	p	95% CI
PM_{10}	(Intercept)	−51.53	32.30	−1.60	0.112	−114.833, 11.769
PM_{10}	Strict Lockdown	6.73	3.65	1.84	0.067	−0.436, 13.891
PM_{10}	Phase 1	7.28	5.50	1.32	0.186	−3.500, 18.066
PM_{10}	Phase 2	16.64	6.40	2.60	**0.010**	4.102, 29.173
PM_{10}	Phase 3	8.44	7.02	1.20	0.230	−5.322, 22.204
PM_{10}	Phase 4	7.79	4.91	1.59	0.114	−1.842, 17.420
PM_{10}	Temperature	3.17	0.80	3.94	**0.001**	1.593, 4.744
PM_{10}	Wind Speed	−3.98	2.46	−1.62	0.107	−8.800, 0.845
PM_{10}	Humidity	0.42	0.25	1.69	0.093	−0.067, 0.902
$PM_{2.5}$	(Intercept)	−10.06	8.39	−1.20	0.232	−26.507, 6.394
$PM_{2.5}$	Strict Lockdown	3.68	0.97	3.80	**0.001**	1.782, 5.578
$PM_{2.5}$	Phase 1	8.12	1.47	5.53	**0.001**	5.242, 10.999
$PM_{2.5}$	Phase 2	7.08	1.70	4.16	**0.001**	3.747, 10.422
$PM_{2.5}$	Phase 3	5.11	1.85	2.76	**0.006**	1.477, 8.750
$PM_{2.5}$	Phase 4	4.17	1.31	3.18	**0.002**	1.598, 6.736
$PM_{2.5}$	Temperature	0.85	0.21	4.06	**0.001**	0.439, 1.259
$PM_{2.5}$	Wind Speed	−2.29	0.66	−3.44	**0.001**	−3.593, −0.986
$PM_{2.5}$	Humidity	0.07	0.07	1.06	0.292	−0.060, 0.199

Pollutant	Predictor	B	SE	t	p	95% CI
NO_2	(Intercept)	3.37	1.51	2.23	**0.026**	0.407, 6.334
NO_2	Strict Lockdown	−1.05	0.17	−6.04	**0.001**	−1.386, −0.707
NO_2	Phase 1	−1.03	0.26	−3.91	**0.001**	−1.549, −0.514
NO_2	Phase 2	−0.96	0.31	−3.13	**0.002**	−1.560, −0.360
NO_2	Phase 3	−0.86	0.33	−2.58	**0.010**	−1.516, −0.207
NO_2	Phase 4	−0.52	0.24	−2.21	**0.028**	−0.982, −0.059
NO_2	Temperature	0.05	0.04	1.39	0.164	−0.021, 0.126
NO_2	Wind Speed	−0.51	0.12	−4.24	**0.001**	−0.743, −0.273
NO_2	Humidity	0.03	0.01	2.43	0.016	0.006, 0.052
O_3	(Intercept)	111.91	8.86	12.62	**0.001**	94.536, 129.284
O_3	Strict Lockdown	−0.04	0.96	−0.04	0.965	−1.924, 1.838
O_3	Phase 1	0.39	1.73	0.22	0.823	−3.008, 3.782
O_3	Phase 2	3.68	2.06	1.79	0.075	−0.356, 7.710
O_3	Phase 3	4.02	2.40	1.67	0.095	−0.689, 8.731
O_3	Phase 4	2.90	2.23	1.30	0.194	−1.464, 7.257
O_3	Temperature	−1.98	0.27	−7.29	**0.001**	−2.514, −1.449
O_3	Wind Speed	1.01	0.61	1.67	0.097	−0.178, 2.207
O_3	Humidity	−0.72	0.06	−12.33	**0.001**	−0.839, −0.609
SO_2	(Intercept)	−0.06	32.75	−0.00	0.998	−64.251, 64.121
SO_2	Strict Lockdown	−0.13	3.50	−0.04	0.971	−6.987, 6.729
SO_2	Phase 1	−3.46	6.36	−0.54	0.587	−15.928, 9.005
SO_2	Phase 2	−7.62	7.56	−1.01	0.314	−22.447, 7.197
SO_2	Phase 3	−9.97	8.82	−1.13	0.260	−27.250, 7.317
SO_2	Phase 4	−11.99	8.92	−1.35	0.180	−29.475, 5.484
SO_2	Temperature	0.73	1.00	0.73	0.464	−1.226, 2.694
SO_2	Wind Speed	−0.74	2.32	−0.32	0.749	−5.298, 3.809
SO_2	Humidity	0.12	0.22	0.56	0.576	−0.302, 0.543

Note. B = unstandardized regression coefficient; SE = standard error; t = t-statistic; p = probability value; 95% CI = 95% confidence interval. Reference category for COVID-19 periods: Pre-Pandemic (1 January–15 March 2020). Meteorological predictors are continuous variables. Significant effects ($p < 0.05$) are indicated in bold.

Table S4. Variable importance from Random Forest models for daily air pollutant concentrations.

Pollutant	Variable	%IncMSE	IncNodePurity	Relative Importance (%)
PM_{10}	COVID-19 Period	11.91	12239.50	31.6
PM_{10}	Temperature	10.29	22239.51	27.3
PM_{10}	Wind Speed	7.77	16837.41	20.6
PM_{10}	Humidity	7.67	21044.93	20.4

Pollutant	Variable	%IncMSE	IncNodePurity	Relative Importance (%)
$PM_{2.5}$	Wind Speed	14.30	1685.86	27.1
$PM_{2.5}$	COVID-19 Period	13.62	1018.20	25.8
$PM_{2.5}$	Humidity	12.47	1434.01	23.6
$PM_{2.5}$	Temperature	12.35	1572.69	23.4
NO_2	COVID-19 Period	20.98	34.45	28.5
NO_2	Humidity	20.08	56.94	27.2
NO_2	Temperature	19.11	53.33	25.9
NO_2	Wind Speed	13.56	46.15	18.4
O_3	Humidity	36.36	2674.53	35.1
O_3	Temperature	31.27	6175.62	30.2
O_3	COVID-19 Period	24.92	5034.00	24.1
O_3	Wind Speed	11.03	1446.58	10.6
SO_2	Temperature	11.17	12762.55	31.8
SO_2	COVID-19 Period	10.40	5912.68	29.6
SO_2	Humidity	9.84	9123.86	28.1
SO_2	Wind Speed	3.67	8876.89	10.5

Note. %IncMSE = percent increase in mean squared error when variable is randomly permuted (higher values indicate greater importance for prediction accuracy); IncNodePurity = total decrease in node impurities from splits on the variable (higher values indicate greater contribution to model fit); Relative Importance = %IncMSE scaled to 100% within each pollutant model. Random Forest models included 500 trees with default node size. Out-of-bag (OOB) error rates ranged from 8.2% (O_3) to 42.3% (NO_2).

Table S5. Model fit and diagnostic statistics for full multiple linear regression models (meteorology + COVID-19 periods).

Pollutant	R^2	Adj. R^2	AIC	BIC	RMSE	df
PM_{10}	0.193	0.174	2788.2	2826.3	15.26	325
$PM_{2.5}$	0.227	0.209	1976.5	2014.9	4.16	335
NO_2	0.257	0.239	804.1	842.6	0.75	338
O_3	0.812	0.806	1458.5	1494.5	3.44	262

Pollutant	R^2	Adj. R^2	AIC	BIC	RMSE	df
SO_2	0.225	0.199	1997.6	2032.9	12.44	242

Note. R^2 = coefficient of determination (proportion of variance explained); Adj. R^2 = adjusted R^2 accounting for number of predictors; AIC = Akaike Information Criterion (lower values indicate better fit); BIC = Bayesian Information Criterion (lower values indicate better fit); RMSE = root mean squared error (in $\mu g/m^3$); df = residual degrees of freedom. All models include three meteorological predictors (temperature, wind speed, humidity) and five COVID-19 period indicators.

Table S6. Comparison of variance partitioning between multiple linear regression and Random Forest approaches for daily air pollutant concentrations in Ilo, Peru.

Pollutant	n	Regression (%)		Random Forest (%)		Pattern
		Meteo	COVID	Meteo	COVID	
**PM_{10}**	334	85.3	14.7	68.4	31.6	Meteorology dominant
**$PM_{2.5}$**	344	52.3	47.7	74.2	25.8	Balanced contributions
**NO_2**	347	57.0	43.0	71.5	28.5	Balanced contributions
**O_3**	271	98.5	1.5	75.9	24.1	Meteorology dominant
**SO_2**	251	93.1	6.9	70.4	29.6	Meteorology dominant

Note. Regression percentages represent the proportion of explained variance (R^2) attributable to meteorological variables (temperature, wind speed, humidity) versus COVID-19 period indicators, calculated via sequential ANOVA. Random Forest percentages represent relative variable importance based on %IncMSE (percent increase in mean squared error when variable is permuted), scaled to 100% within each pollutant model. Pattern classification: "Meteorology dominant" indicates >70% contribution from meteorological variables in regression analysis; "Balanced contributions" indicates 40-60% from each factor group. Despite methodological differences, both approaches consistently identify O_3 as meteorology-driven and $PM_{2.5}/NO_2$ as showing substantial COVID-19 restriction effects.

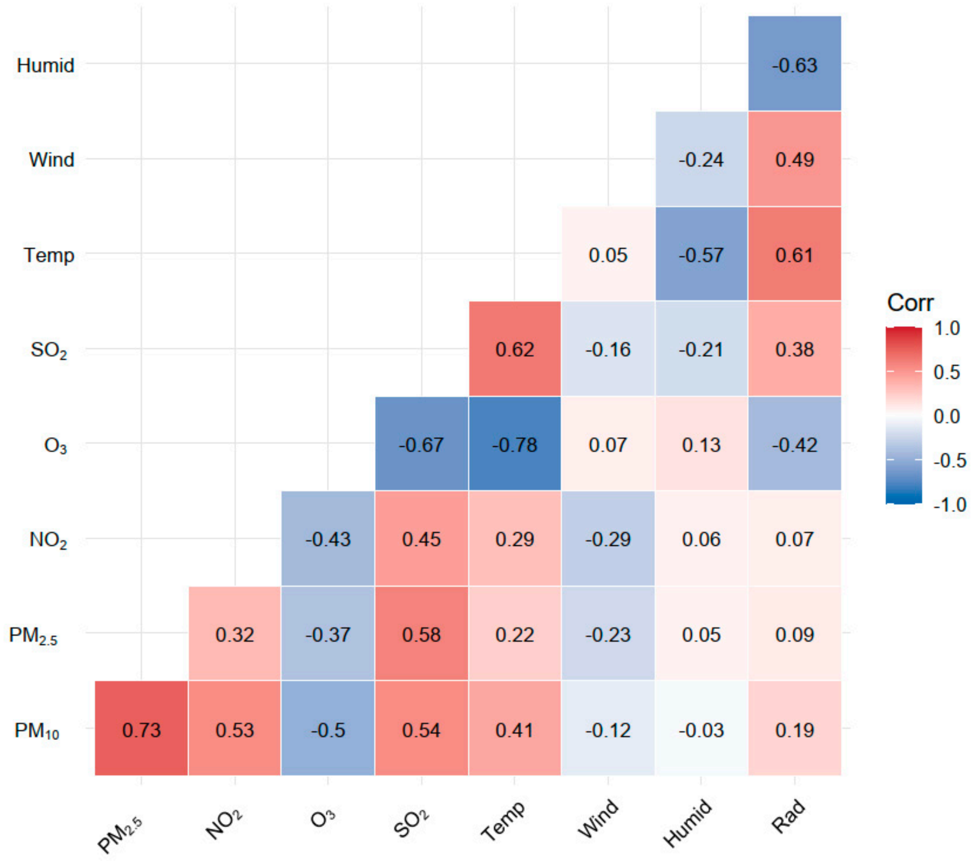

Figure S1. Correlation matrix between daily air pollutant concentrations and meteorological variables in Ilo, Peru. Heatmap of Spearman rank correlation coefficients (ϱ) based on n = 238 daily observations for PM_{10}, $PM_{2.5}$, NO_2, O_3, SO_2, temperature, wind speed, relative humidity and solar radiation. Only the lower triangle of the matrix is displayed for clarity. Cell colours range from blue (negative correlation) to red (positive correlation), with intensity proportional to the absolute value of ϱ; numeric coefficients are shown within cells. The matrix reveals, for example, strong positive associations between PM_{10} and $PM_{2.5}$ and between temperature and solar radiation, and strong negative associations between O_3 and NO_2 and between temperature and humidity. These correlation patterns informed the selection of covariates in the multiple regression models and underscore the need to account for meteorological confounding when assessing COVID-19 restriction effects on air quality.

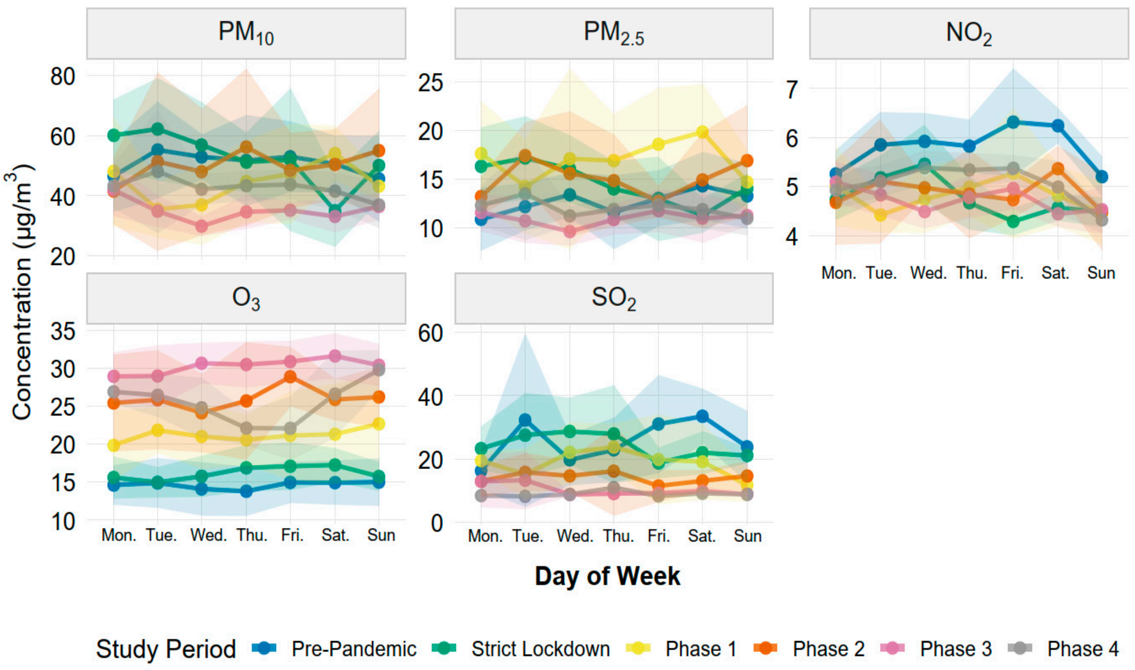

Figure S2. Daily mean concentrations of PM_{10}, $PM_{2.5}$, NO_2, O_3 and SO_2 are shown by day of week during the COVID-19 pandemic in Ilo, Peru. Mean daily concentrations of PM_{10}, $PM_{2.5}$, NO_2, O_3 and SO_2 are shown by day of week (Monday–Sunday) and study phase (Pre-Pandemic, Strict Lockdown, and Phases 1–4). Lines connect daily means and shaded bands represent 95% confidence intervals. Colours follow the conventions used in Figure 4. The panels illustrate how weekday–weekend contrasts evolved across phases, with clearer weekly modulation for traffic-related pollutants (e.g., NO_2) than for particulate matter, and provide complementary evidence on the influence of mobility and industrial activity patterns beyond the initial lockdown.

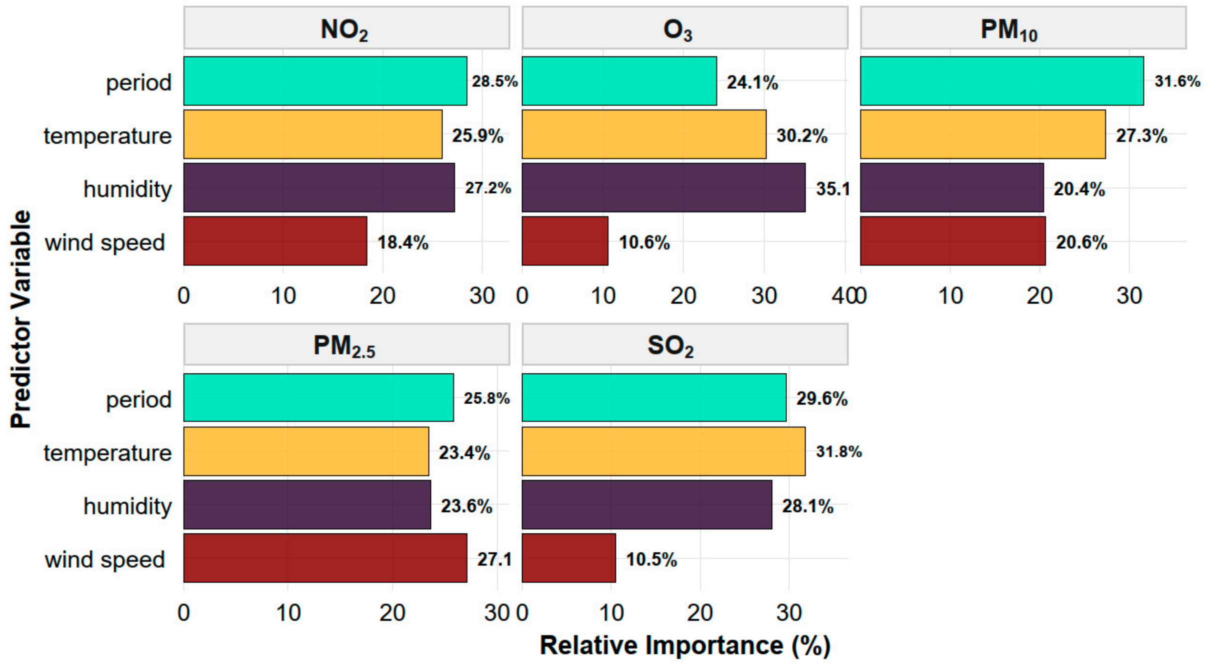

Figure S3. Relative importance of COVID-19 period and meteorological predictors for daily air pollutant concentrations in Ilo, Peru. Horizontal bar plots show variable importance from random forest models for PM_{10}, $PM_{2.5}$, NO_2, O_3 and SO_2. Predictor variables are COVID-19 period (cyan), temperature (orange), relative humidity (purple) and wind speed (red). Importance is expressed as the percentage increase in mean squared error (%IncMSE) when each variable is randomly permuted, and values are rescaled to sum to 100% within each pollutant. Higher percentages indicate stronger contribution of the predictor to model performance. These patterns provide a non-linear complement to the multiple regression variance partitioning (Figure 8), supporting the relative roles of COVID-19 restriction periods and meteorological conditions in explaining pollutant variability.

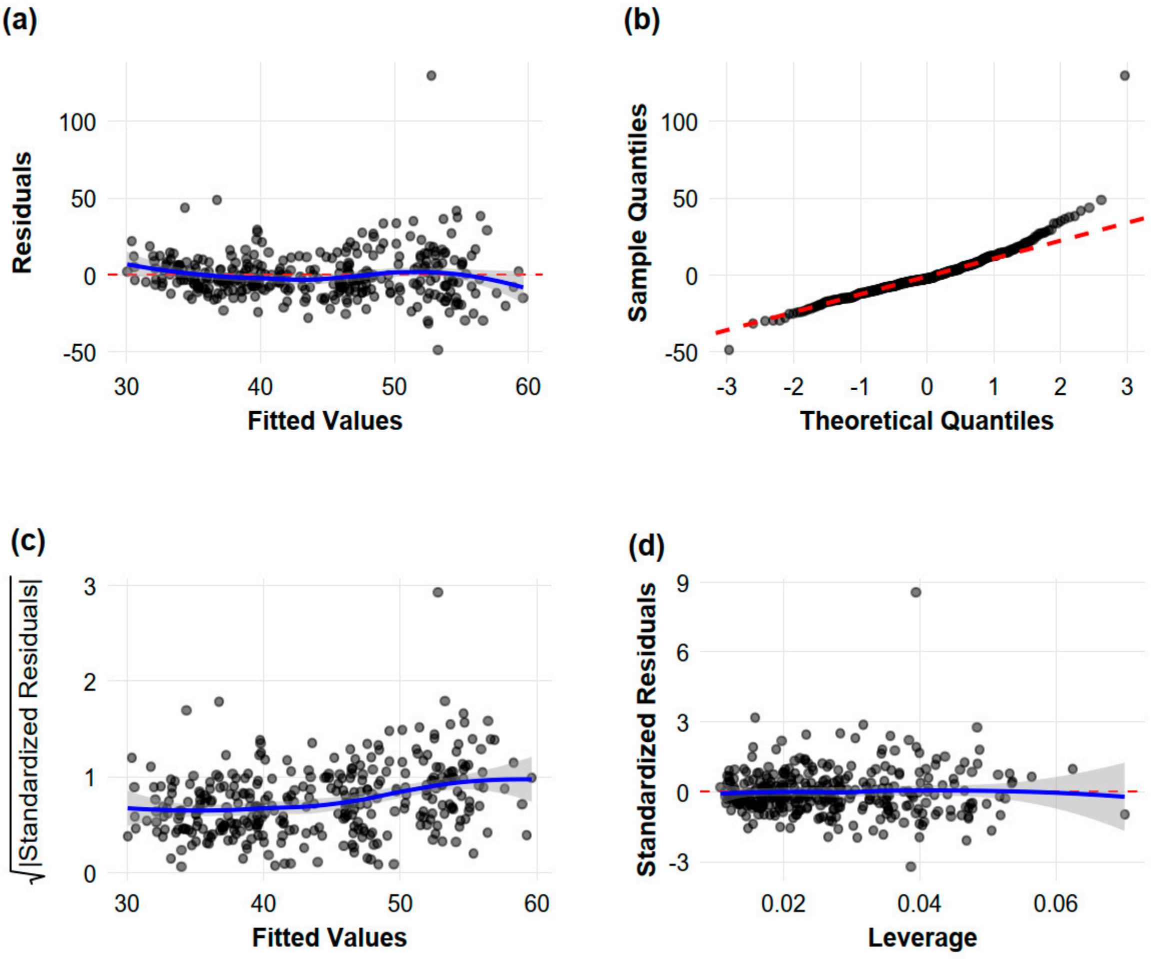

Figure S4. Diagnostic plots for the multiple linear regression model of daily PM_{10} concentrations in Ilo, Peru. Panels (a–d) display standard regression diagnostics for the PM_{10} model, representative of all pollutant models. (a) Residuals versus fitted values, used to assess linearity and homoscedasticity. (b) Normal Q–Q plot of standardized residuals, used to evaluate approximate normality. (c) Scale–location plot showing the square root of standardized residuals versus fitted values, assessing constancy of variance. (d) Standardized residuals versus leverage, used to identify observations with high leverage and potential influence. The absence of pronounced trends or extreme points in these plots indicates

that model assumptions are reasonably satisfied. Similar diagnostic patterns were obtained for $PM_{2.5}$, NO_2, O_3 and SO_2 models (not shown).

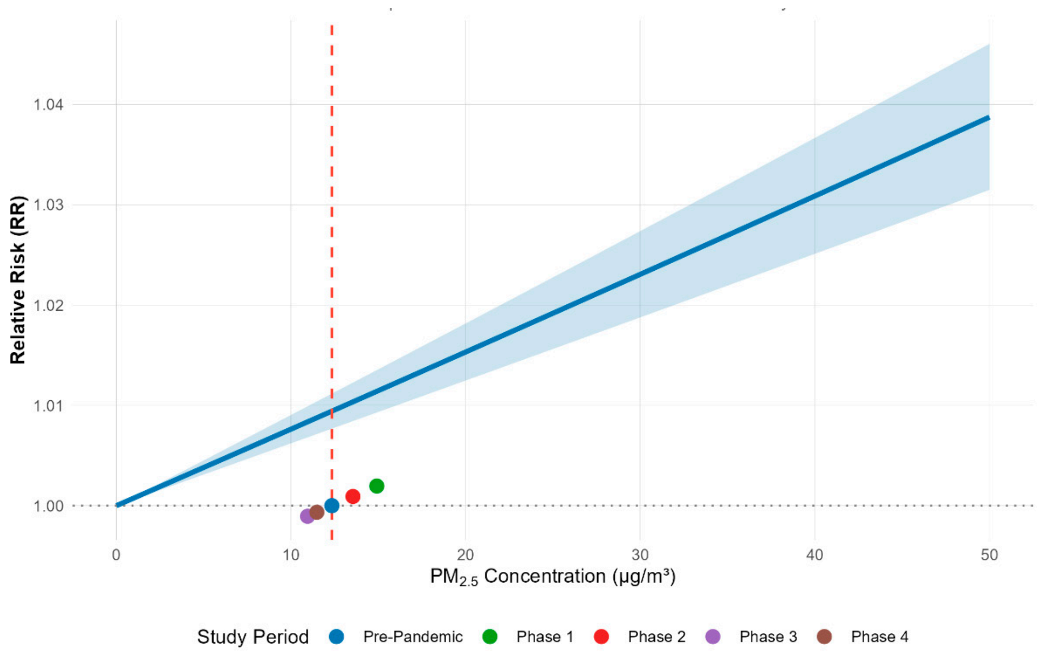

Figure S5. Concentration–response relationship between long-term $PM_{2.5}$ exposure and cardiovascular mortality. The solid black curve depicts the relative risk (RR) of cardiovascular mortality as a function of annual mean $PM_{2.5}$ concentration (µg/m^3), based on the integrated exposure–response (IER) function proposed by Burnett et al. [58] and adopted in the GBD 2019 study [60]. The shaded band represents the corresponding 95% confidence interval. Coloured points show the pre-pandemic baseline and each COVID-19 phase (Pre-Pandemic and Phases 1–4), following the colour scheme used in the main-text figures. The vertical dashed line marks the pre-pandemic mean $PM_{2.5}$ concentration in Ilo, and the horizontal dashed line indicates RR = 1.0 (no excess risk). The position of the phase-specific points along the curve illustrates how relatively small changes in the 10–20 µg/m^3 range can translate into measurable differences in cardiovascular risk.
